# Mutant KRAS‐m^6^A Epitranscriptome Axis Promotes Colorectal Cancer and is a Therapeutic Target

**DOI:** 10.1002/advs.77627

**Published:** 2026-09-09

**Authors:** Danyu Chen, Pingmei Huang, Ruizhi Tao, Fenfen Ji, Qinyao Wei, Lok Hin Ko, Lianxin Zhou, Huarong Chen, Alvin Cheung, Wei Kang, Ko Fai To, Jun Yu, Chi Chun Wong

**Affiliations:** ^1^ Institute of Digestive Disease and Department of Medicine and Therapeutics State Key Laboratory of Digestive Disease Li Ka Shing Institute of Health Sciences The Chinese University of Hong Kong Hong Kong Hong Kong SAR China; ^2^ Department of Anaesthesia Intensive Care and Peter Hung Pain Research Institute The Chinese University of Hong Kong Hong Kong Hong Kong SAR China; ^3^ Department of Anatomical and Cellular Pathology State Key Laboratory of Translational Oncology Prince of Wales Hospital The Chinese University of Hong Kong Hong Kong Hong Kong SAR China

**Keywords:** autophagy, biology, cancer research, cd34, gene mutation, kras, mutant, tumor microenvironment

## Abstract

The interaction between gene mutations and epitranscriptomic alterations in colorectal cancer (CRC) remains poorly understood. Here, we identified that KRAS mutations, present in ∼40%–50% of CRC patients, act as driver mutations mediating an aberrant epitranscriptome. In human CRC patients, mutant KRAS correlates with increased m^6^A mRNA modification. In isogenic CRC cells and animal models expressing wild‐type KRAS or mutant KRAS, m^6^A modification was upregulated in mutant KRAS cells/mice. Mutant KRAS promotes m^6^A modification by stabilizing METTL3 protein, an m^6^A writer, through inhibiting its degradation via p62‐driven selective autophagy. Integrative RNA‐seq, m^6^A‐seq, and RIP‐seq revealed BCL9L as a target of mutant KRAS‐mediated m^6^A. Mutant KRAS promotes METTL3‐dependent BCL9L m^6^A modification and YTHDF1‐dependent translation, leading to elevated BCL9L translation and protein expression. Mechanistically, the METTL3‐m^6^A‐BCL9L axis promotes secretion of TGF‐β1/β2/β3 to enrich immunosuppressive T_reg_ in the tumor microenvironment. BCL9L knockout thus significantly impaired KRAS‐mutant CRC growth in murine allograft models and human xenografts in CD34^+^ immunohumanized mice, concomitant with T_reg_ suppression and Th1 activation. Finally, we demonstrated that pharmacological blockade of METTL3 by STC‐15 or STM2457 synergized with mutant KRAS inhibitors to inhibit KRAS‐mutant CRC growth in mouse models, indicating a promising strategy to boost the efficacy of KRAS inhibitors in KRAS‐mutant CRC.

## Graphical abstract

Mutant KRAS‐m^6^A axis promotes colorectal cancer (CRC) progression. Mutant KRAS stabilizes METTL3, leading to increased m^6^A‐modified BCL9L mRNA and translation of BCL9L protein. BCL9L in turn mediates TGF‐β secretion to induce differentiation of T_reg_ and an immunosuppressive microenvironment. Co‐targeting mutant KRAS and METTL3 synergistically suppressed the growth of KRAS‐mutant CRC.

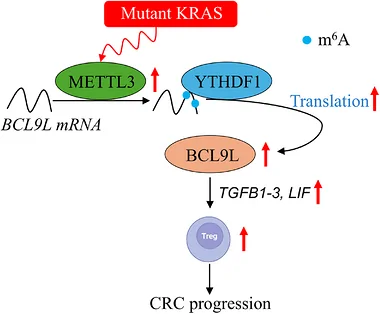

## 1 Introduction

Colorectal cancer (CRC) is one of the top cancers worldwide, and a leading cause of cancer‐related deaths [1]. In spite of the advances in chemotherapy and molecularly targeted therapy, CRC patients with advanced or metastatic disease often suffer from poor prognosis, with a 5‐year survival of less than 12% [2, 3]. Colorectal tumorigenesis is initiated by the inactivation of APC (90%), a tumor suppressor. Mutant KRAS represents the second hit (40%–50%) that drives colorectal adenoma and carcinoma formation [4, 5]. Not only that, KRAS mutations mediate primary resistance to anti‐EGFR treatment in CRC patients [6], and have been associated with non‐response to immune checkpoint blockade (ICB) therapy [7, 8]. The targeting of KRAS‐mutant CRC represents an unmet medical need.

Whilst it is recognized early on that KRAS is a pivotal oncogenic factor in CRC, the targeting of KRAS represents a major therapeutic challenge. Indeed, for decades KRAS was considered “undruggable” [9]. The approval of the first‐in‐class KRAS^G12C^ inhibitors, e.g., Sotorasib in 2021 for KRAS‐mutant lung cancer, represents a breakthrough in targeting KRAS in cancer [10, 11]. Inhibitors targeting alternative KRAS isoforms (e.g. KRAS^G12D^) are emerging in the drug pipeline [12], as are pan‐mutant KRAS [13] and pan‐mutant RAS blockers [14]. However, despite the clinical success of KRAS^G12C^ inhibitors in lung and pancreatic cancers [15, 16], response rates in CRC have been disappointing (< 10%) [17]. Notably, drug combinations have been shown to be the way forward to target KRAS‐mutant CRC [18], as exemplified by the recent approval of Sotorasib plus Panitumumab in KRAS^G12C^ CRC [19]. Nevertheless, therapeutic resistance remains a persistent challenge, even in the context of dual targeting of KRAS and EGFR [20]. Thus, the development of novel approaches that can boost responsiveness to KRAS inhibitors in CRC is urgent and important.

CRC involves the co‐operative action of genetic, epigenetic, and epitranscriptomic alterations. RNA N^6^‐methyladenosine (m^6^A) is the most abundant mRNA modification in humans, and it controls mRNA splicing, mRNA stability, and translation efficiency. M^6^A is tightly regulated by m^6^A modification enzymes, including m^6^A writers (METTL3) and erasers (ALKBH5). M^6^A modification facilitates their interaction with m^6^A readers (YTHD and IGF2BP family), which in turn determines the consequential fate of mRNA. M^6^A plays a decisive role in cancer by promoting cell proliferation and stemness, and modulating the tumor microenvironment [21, 22, 23, 24]. Of particular interest, drug candidates specifically targeting METTL3 regulators have emerged from various drug pipelines [25, 26]. However, the potential interactions between mutant KRAS and m^6^A modification remain unexplored.

In this study, we reveal the unreported association between mutant KRAS and m^6^A in CRC. Mutant KRAS in CRC patients, animal models and CRC cell lines is associated with elevated m^6^A modification, accompanied by the upregulation of m^6^A writer METTL3. Mutant KRAS confers stability to the METTL3 protein by suppressing its autophagy‐mediated degradation. We further demonstrated BCL9L as a focal point of the mutant KRAS‐METTL3 axis and that BCL9L promotes CRC by inducing T_reg_ expansion. Finally, pharmacological targeting of mutant KRAS and METTL3 synergized to suppress CRC, implying a promising therapeutic modality for KRAS‐mutant CRC.

## 2 Results

### 2.1 Mutant KRAS Promotes m6A mRNA Modification In Vitro, In Vivo and in Human CRC

**FIGURE 1:**
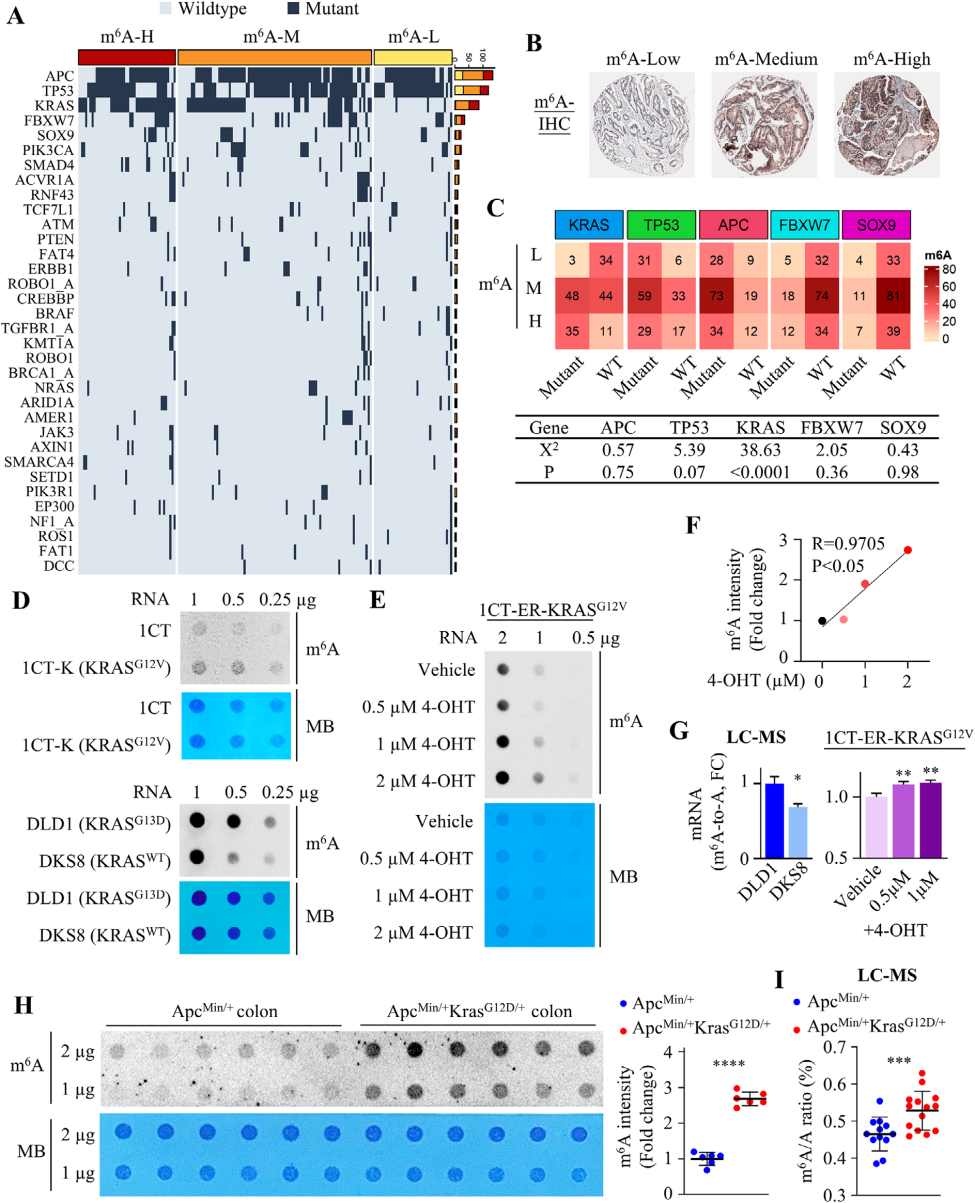
M^6^A modification is elevated in KRAS mutant‐CRC patients, cell lines and mice (A) Distribution of cancer‐associated genetic mutations and m^6^A immunohistochemistry scores in a tissue microarray cohort of CRC patients (N = 175). M^6^A staining intensity was scored by a pathologist blinded to sample identity into High (H), Medium (M) and Low (L). (B) Representative m^6^A IHC staining of the tissue microarray cohort. (C) Correlation analyses between cancer‐associated genetic mutations and m^6^A scores using a chi‐squared test. (D) Dot blot analysis of mRNA m^6^A levels in 1CT, 1CT‐K (KRAS^G12V^), DLD1 (KRAS^G13D^), and DKS8 (KRAS^WT^) cell lines using anti‐m^6^A antibody. Total mRNA loading was determined by methylene blue staining. (E) Dot blot analysis of mRNA m^6^A levels in 1CT‐ER‐KRAS^G12V^ treated with vehicle or 0.5–2 µm 4‐hydroxytamoxifen (4‐OHT) for 72 h using anti‐m^6^A antibody. Total mRNA loading was determined by methylene blue staining. (F) Dose‐dependent effect of 4‐OHT on m^6^A intensity, as determined by dot blot analysis. (G) LC‐MS/MS analysis of m^6^A modification in mRNA from cell lines. Total mRNA extracted from cells was digested by nucleases, followed by LC‐MS/MS assay. Student's T‐test. (H) Dot blot of mRNA m^6^A levels in colon tissues of Apc^Min/+^Kras^G12D/+^ and Apc^Min/+^mice. Total mRNA loading was determined by methylene blue staining. Student's T‐test. (I) LC‐MS/MS analysis of m^6^A modification in mRNA in colon tissues of Apc^Min/+^Kras^G12D/+^ and Apc^Min/+^mice. Student's T‐test.

To evaluate whether genetic mutations are associated with m^6^A modification in human CRC, we utilized a tissue microarray of 175 CRC patients from Hong Kong. The overall m^6^A levels were detected by an anti‐m^6^A antibody, followed by histological evaluation into three classes (low, medium, and high) (Figure 1A,B). We then determined the correlation of m^6^A staining and genetic mutations based on the targeted sequencing of 97 cancer‐associated genes (Figure 1A). Across 97 genetic mutations, we observed that m^6^A‐high CRC patients have a markedly high frequency of KRAS mutations compared to m^6^A‐low counterparts. Concordantly, among all the prevalent genetic mutations in CRC (APC, TP53, KRAS, FBXW7, and SOX9), only mutations in KRAS significantly correlated with m^6^A staining intensity (*P* < 0.0001, X^2^ = 38.6) (Figure 1C). These data indicate that KRAS mutations might have a positive impact on m^6^A modification in CRC patients.

To examine whether KRAS mutation influenced the epitranscriptome, we employed three pairs of isogenic cell lines: 1) DLD1 (KRAS^G13D/+^) and DKS8 (KRAS^+/−^) cells, with knockout of the mutant KRAS allele; 2) normal colonic 1CT and 1CT‐KRAS^G12V^ cells with ectopic expression of mutant KRAS; 3) 1CT‐ER‐KRAS^G12V^ cells, which overexpress an inducible mutant ER‐KRAS that is activated by 4‐hydroxytamoxifen (4‐OHT) addition. We first performed a dot blot assay to evaluate total m6A levels in mRNA. The overexpression of mutant KRAS^G12V^ in 1CT cells increased m^6^A levels (Figure 1D), whereas mutant KRAS knockout in DKS8 suppressed m^6^A levels compared to DLD1 cells (Figure 1D). We next incubated 1CT‐ER‐KRAS^G12V^ cells with escalating 4‐OHT doses (Figure 1E), revealing that 4‐OHT dose‐dependently increased m^6^A levels (*R* = 0.9705, *P* < 0.05) (Figure 1F). To confirm these results, we performed targeted LC‐MS to determine the m^6^A‐to‐A ratio in mRNA. As shown in Figure 1G, DKS8 cells with knockout of mutant KRAS showed decreased m^6^A compared to DLD1 cells, whereas the activation of mutant KRAS in 1CT‐ER‐KRAS^G12V^ cells increased m^6^A levels, implying that mutant KRAS promotes m^6^A mRNA modification in vitro.

To corroborate our observations in vivo, we examined m^6^A modification levels in *Apc* ^Min/+^ and *Apc* ^Min/+^ *Kras* ^G12D/+^ *Villin*‐*Cre* mouse colon tissues by dot blot, demonstrating a consistent increase in mRNA m^6^A levels in *Apc* ^Min/+^ *Kras* ^G12D/+^ *Villin*‐*Cre* mice (*P* < 0.0001; Figure 1H). Targeted LC‐MS analysis confirmed increased m^6^A‐to‐A ratios in the *Apc* ^Min/+^ *Kras* ^G12D/+^ *Villin*‐*Cre* mouse colon compared to *Apc* ^Min/+^ counterparts (*P* < 0.001; Figure 1I). Collectively, studies in isogenic cell lines and mice harboring mutant KRAS implicate its function in promoting m^6^A modification in CRC.

### 2.2 Mutant KRAS Promotes METTL3 Expression

**FIGURE 2:**
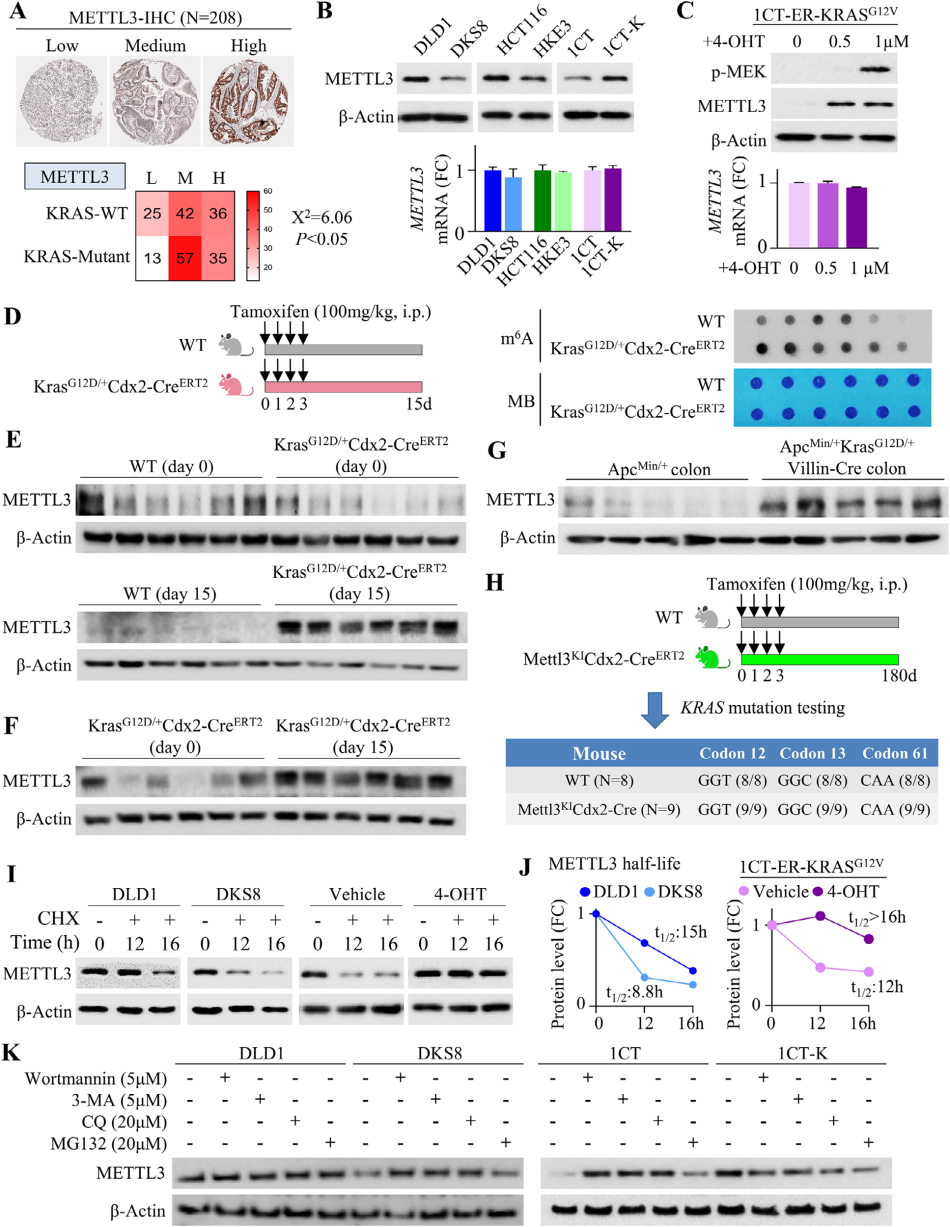
METTL3 protein expression is increased in KRAS‐mutant CRC patients, cell lines and mice (A) METTL3 protein was determined by immunohistochemistry in a tissue microarray cohort of CRC patients (N = 208). Representative METTL3 IHC staining (*upper*). Correlation analyses between METTL3 and KRAS mutation using a chi‐squared test. (B) METTL3 protein and mRNA expression in 1CT, 1CT‐K (KRAS^G12V^), DLD1 (KRAS^G13D^), and DKS8 (KRAS^WT^), HCT116 (KRAS^G13D^), and HKE3 (KRAS^WT^) cell lines, as determined by western blot and qPCR. (C) METTL3 protein and mRNA expression in 1CT‐ER‐KRAS^G12V^ treated with vehicle, 0.5 or 1 µm 4‐hydroxytamoxifen (4‐OHT) for 72 h, as determined by western blot and qPCR. (D) Conditional, intestine‐specific KRAS^G12D^ model. Kras^G12D/+^Cdx2‐Cre^ERT2^ mice and their wildtype littermates were given four tamoxifen injections (100 mg/kg, i.p.). Mouse colon was harvested at baseline or 15 days after the initial tamoxifen dose (*left*). Dot blot analysis of mRNA m^6^A levels in the colon tissues from Kras^G12D/+^Cdx2‐Cre^ERT2^ and wildtype mice. (E) METTL3 expression in Kras^G12D/+^Cdx2‐Cre^ERT2^ and wildtype mice on baseline (*upper*) and day 15 after mutant KRAS activation (*lower*), as determined by western blot. (F) METTL3 expression in Kras^G12D/+^Cdx2‐Cre^ERT2^ mice on baseline and day 15 after mutant KRAS activation, as determined by western blot. (G) METTL3 expression in colon tissues of Apc^Min/+^Kras^G12D/+^ and Apc^Min/+^mice, as evaluated by western blot. (H) Conditional, intestine‐specific METTL3 knock‐in model. Mettl3^KI^Cdx2‐Cre^ERT2^ and their wildtype littermates received four tamoxifen injections (100 mg/kg, i.p.) and were followed up for 180 days. Thereafter, colon tissues were extracted and subjected to KRAS mutation analysis by Sanger sequencing. (I) METTL3 protein stability in DLD1 (KRAS^G13D^), DKS8 (KRAS^WT^), and 1CT‐ER‐KRAS^G12V^ cells treated with vehicle or 4‐OHT. The cells were treated with cycloheximide for the indicated time points. (J) Half‐lives of METTL3 protein in different cell lines, as evaluated by densitometry analysis of the western blot data in Figure 2I. (K) Effect of autophagy inhibitors (Wortmannin, 3‐MA: 3‐Methyladenine, CQ: Chloroquine) and proteasome inhibitor MG132 on METTL3 protein expression in 1CT, 1CT‐K (KRAS^G12V^), DLD1 (KRAS^G13D^), and DKS8 (KRAS^WT^) cells. Cells were treated with these inhibitors for 24 h prior to harvesting.

Cellular mRNA m^6^A modification is catalyzed by the m^6^A methyltransferase complex, where METTL3 functions as the catalytic subunit. We thus determined METTL3 expression with respect to mutant KRAS in our tissue microarray cohort (Figure 2A), showing KRAS‐mutant CRC patients had significantly higher METTL3 scores as compared to KRAS‐wildtype CRC patients (*P* < 0.05, X^2^ = 6.06). To ask if the regulation of m^6^A regulatory enzymes is modulated by mutant KRAS at the transcriptional level, we determined METTL3 mRNA expression in isogenic cell line pairs with or without mutant KRAS (Figure 2B). However, no significant differences were found for METTL3 in isogenic KRAS knockout (DLD1 vs. DKS8; HCT116 vs. HKE3) and KRAS overexpressing cells (1CT vs. 1CT‐K). Next, we evaluated METTL3 protein expression by western blot (Figure 2B). KRAS‐mutant knockout reduced METTL3 protein levels. Conversely, the overexpression of mutant KRAS^G12V^ in 1CT‐K cells elevated METTL3 protein compared to 1CT cells (Figure 2B). Consistently, the addition of 4‐OHT to 1CT‐ER‐KRAS^G12V^ cells had no effect on METTL3 mRNA levels (Figure 2C), but increased its protein levels with an even stronger response compared to p‐MEK (Figure 2C), the major effector kinase of the KRAS‐MEK‐ERK pathway, suggesting that KRAS mutation has a profound effect in up‐regulating METTL3 expression.

To validate that mutant KRAS regulates METTL3 expression in vivo, we generated an intestine‐specific, conditional *Kras* ^G12D/+^ *Cdx2‐Cre* ^ERT2^ knock‐in model (Figure 2D). Colon tissues were collected at baseline and day 15 after the initial tamoxifen injection. Consistently, knock‐in of mutant KRAS increased m^6^A levels after 15 days compared to wildtype littermates (Figure 2D). Compared to wildtype mice, colon METTL3 protein expression was markedly increased in *Kras* ^G12D/+^ *Cdx2‐Cre* ^ERT2^ mice on day 15 (Figure 2E). No significant difference was observed in baseline colon, although the sample size might limit the ability to detect small differences in METTL3 protein (Figure 2E). METTL3 mRNA was not significantly different between *Kras* ^G12D/+^ *Cdx2‐Cre* ^ERT2^ mice and wildtype littermates at baseline and on day 15 (Figure S1). As expected, METTL3 protein was induced in *Kras* ^G12D/+^ *Cdx2‐Cre* ^ERT2^ mice on day 15 compared to baseline (Figure 2F), implying that short‐term mutant KRAS activation is sufficient to drive METTL3 protein expression. Confirming METTL3 up‐regulation by mutant KRAS in CRC, METTL3 protein expression was higher in *Apc* ^Min/+^ *Kras* ^G12D/+^ *Villin*‐*Cre* mouse colon tissues compared to *Apc* ^Min/+^ counterparts (Figure 2G). We asked if METTL3 can reciprocally modulate KRAS status. However, intestine‐specific METTL3 knock‐in failed to alter KRAS mutational status in mice (Figure 2H). Together, these data suggest that mutant KRAS promotes METTL3 protein expression in vitro and in vivo.

### 2.3 Mutant KRAS Stabilizes METTL3 by Inhibiting Its Degradation via Autophagy

We next sought to understand how mutant KRAS regulates METTL3 protein expression. As mutant KRAS had no effect on METTL3 mRNA levels, we asked if mutant KRAS affected protein stability. To this end, we measured METTL3 protein degradation after the inhibition of protein synthesis by cycloheximide (CHX). METTL3 protein stability was reduced in DKS8 compared to DLD1 cells (Figure 2I). In contrast, 4‐OHT‐induced mutant KRAS in 1CT‐ER‐KRAS^G12V^ cells stabilized METTL3 protein expression as compared to vehicle (Figure 2I). Correspondingly, the half‐life of METTL3 was reduced in DKS8 cells compared to DLD1 cells, whereas the addition of 4‐OHT in 1CT‐ER‐KRAS^G12V^ cells exerted an opposite effect (Figure 2J). These results indicate that mutant KRAS protects METTL3 from protein degradation.

Protein degradation is mediated by two main pathways: ubiquitin‐proteasome and autophagy‐lysosomal pathways. To pinpoint the underlying mechanism involved in METTL3 degradation, we next treated isogenic cell lines with inhibitors of the proteasome (MG132) or autophagy (wortmannin; 3‐methyladenine, 3‐MA; chloroquine, CQ). We found that autophagy inhibitors rescued METTL3 protein expression in KRAS‐wildtype cells, while they had no effect on isogenic KRAS‐mutant cells (Figure 2K), and MG132 had no effect on METTL3 protein expression in either KRAS‐wildtype or mutant cell lines (Figure 2K). Consistently, CQ restored protein expression of METTL3 in 1CT‐ER‐KRAS^G12V^ cells treated with vehicle to levels comparable to that of 4‐OHT‐treated cells (Figure S2).

**FIGURE 3:**
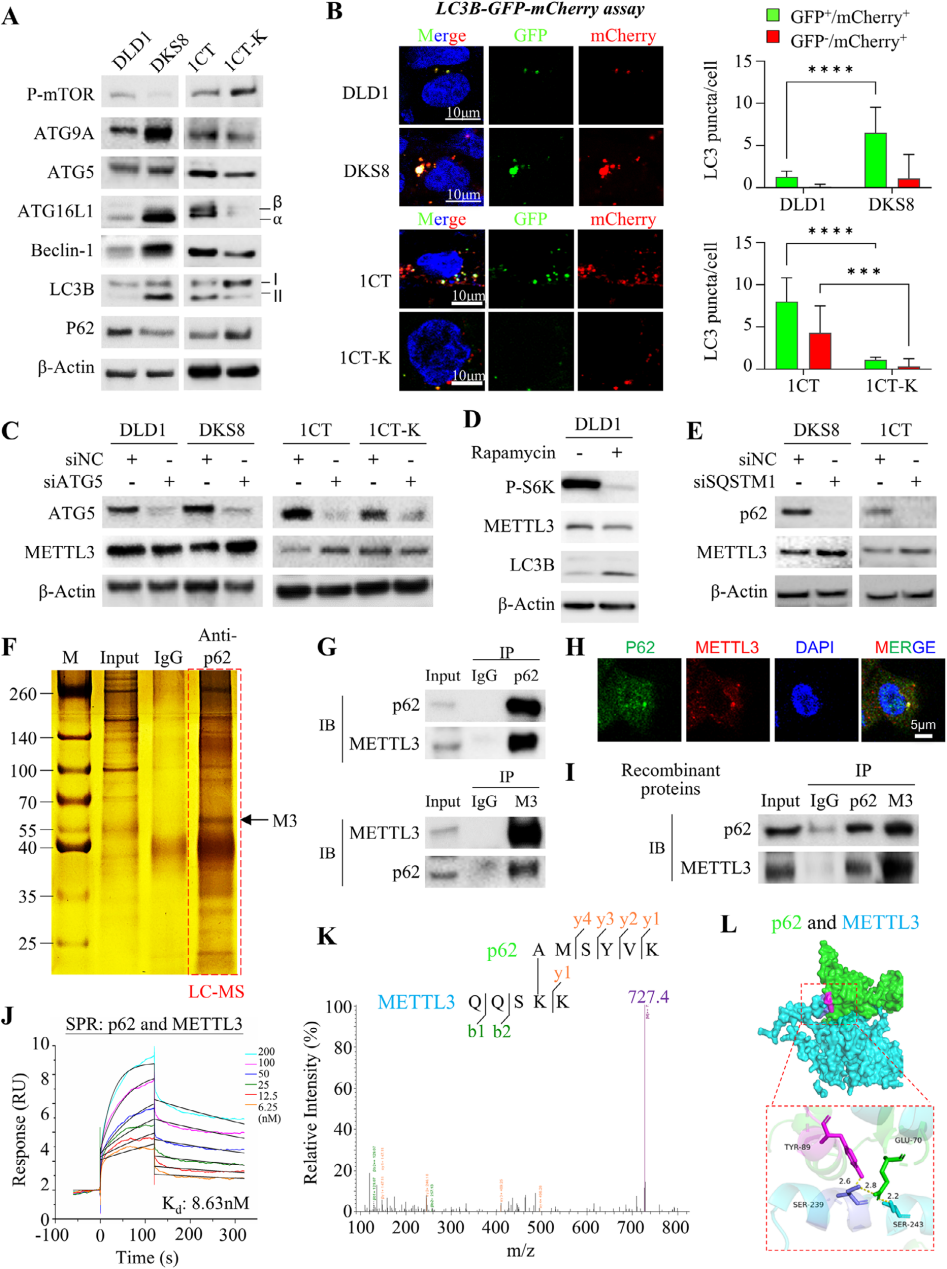
Mutant KRAS promotes METTL3 protein expression by inhibiting autophagy‐mediated degradation (A) Protein expression of autophagy markers in 1CT, 1CT‐K (KRAS^G12V^), DLD1 (KRAS^G13D^), and DKS8 (KRAS^WT^) cells. (B) Autophagy flux in 1CT, 1CT‐K (KRAS^G12V^), DLD1 (KRAS^G13D^), and DKS8 (KRAS^WT^) cells. Cells were transfected with the LC3B‐GFP‐mCherry plasmid for 48 h, followed by confocal microscopy analysis. Student's T‐test. (C) Effect of ATG knockdown on METTL3 protein expression in 1CT, 1CT‐K (KRAS^G12V^), DLD1 (KRAS^G13D^), and DKS8 (KRAS^WT^) cells. (D) Effect of autophagy induction by rapamycin on METTL3 protein expression in DLD1 cells (0.1 µm, 48 h). (E) Effect of p62 (SQSTM1) knockdown on METTL3 protein expression in 1CT and DKS8 (KRAS^WT^) cells. (F) Anti‐p62 pulldown using DLD1 cell lysates, followed by band excision and LC‐MS mass spectrometry‐based protein identification. METTL3 was a top‐enriched protein. (G) Pulldown assay using anti‐p62 (*upper*) and anti‐METTL3 (*lower*) in DLD1 cells, leading to the enrichment of METTL3 and p62, respectively. (H) Co‐immunofluorescence assay of METTL3 (Red) and p62 (Green), followed by confocal microscopy analysis. (I) Pulldown assays using IgG, anti‐p62 or anti‐METTL3 with recombinant METTL3 and p62 proteins. (J) Surface Plasmon Resonance (SPR) assay of the interaction between p62 and METTL3. p62 was immobilized, and then different concentrations of METTL3 were added sequentially to the reaction. (K) Cross‐linking mass spectrometry analysis identified the interacting domain and amino acid residues between METTL3 and p62. (L) In silico molecular docking analysis of p62 and METTL3 proteins. ^*^, *p* < 0.05; ^**^, *p* < 0.01; ^***^, *p* < 0.001; ^****^, *p* < 0.0001.

To examine the impact of KRAS on autophagy, we performed western blot and demonstrated that the knockout of mutant KRAS downregulated p‐mTOR and up‐regulated key autophagic markers, including ATG9A, ATG5, ATG16L1, and Beclin‐1, together with the cleavage of LC3B (Figure 3A). Conversely, mutant KRAS overexpression in 1CT cells exerted opposite effects (Figure 3A). To evaluate changes in autophagic flux, we transfected isogenic cell lines with the mCherry‐GFP‐LC3B vector and performed confocal microscopy (Figure 3B). Compared to DLD1 cells, DKS8 cells exhibited a significant increase in the number of autophagosomes (mCherry^+^GFP^+^); on the contrary, 1CT‐K cells expressing mutant KRAS had downregulated autophagosomes and autolysosomes (mCherry^+^GFP^−^) (Figure 3B), inferring that mutant KRAS reduced autophagic flux in CRC cells. Mutant KRAS thus stabilized METTL3 expression by inhibiting METTL3 protein degradation via the autophagy‐lysosomal pathway.

### 2.4 p62 Functions as a Receptor for METTL3 and YTHDF1 in CRC Cells

To identify the components involved in autophagic degradation of METTL3, we performed knockdown of ATG5, involved in autophagosome formation. ATG5 siRNA also rescued METTL3 protein expression in KRAS‐wildtype cells (Figure 3C). Autophagy induction with the mTOR inhibitor Rapamycin reduced METTL3 protein expression (Figure 3D), although this cannot exclude autophagy‐independent effects of mTOR blockade, such as proteasomal degradation or translational regulation. p62 is an autophagy receptor that targets client proteins to the autophagosome. p62 knockdown also restored METTL3 expression in KRAS‐wildtype DKS8 and 1CT cells (Figure 3E), implying that p62 might serve as a receptor. Next, we performed a p62 pulldown assay, followed by mass spectrometry for protein identification, revealing that METTL3 as a top candidate co‐immunoprecipitated by anti‐p62 (Figure 3F). Co‐immunoprecipitation (co‐IP) using anti‐p62 and anti‐METTL3 confirmed the interaction between p62 and METTL3 (Figure 3G). In situ immunofluorescence (IF) staining identified co‐localization of p62 signals and METTL3 in the cytosolic fraction of CRC cells (Figure 3H). Co‐IP assays utilizing recombinant p62 and METTL3 proteins validated direct protein‐protein interaction between the two (Figure 3I). Surface plasmon resonance (SPR) assay demonstrated that p62 physically interacts with METTL3, with a K_d_ of 8.63 nm (Figure 3J). Crosslinking mass spectrometry and molecular docking revealed binding residues at the p62‐METTL3 interface (Figure 3K,L). Hence, p62 is an autophagy receptor for METTL3, and it facilitates METTL3 degradation via the autophagy‐lysosomal pathway. This process is inhibited by mutant KRAS in CRC cells, thus promoting the stabilization of METTL3 protein.

### 2.5 Multiomic Analysis Unraveled BCL9L as a Downstream Target of Mutant KRAS‐m6A Axis

**FIGURE 4:**
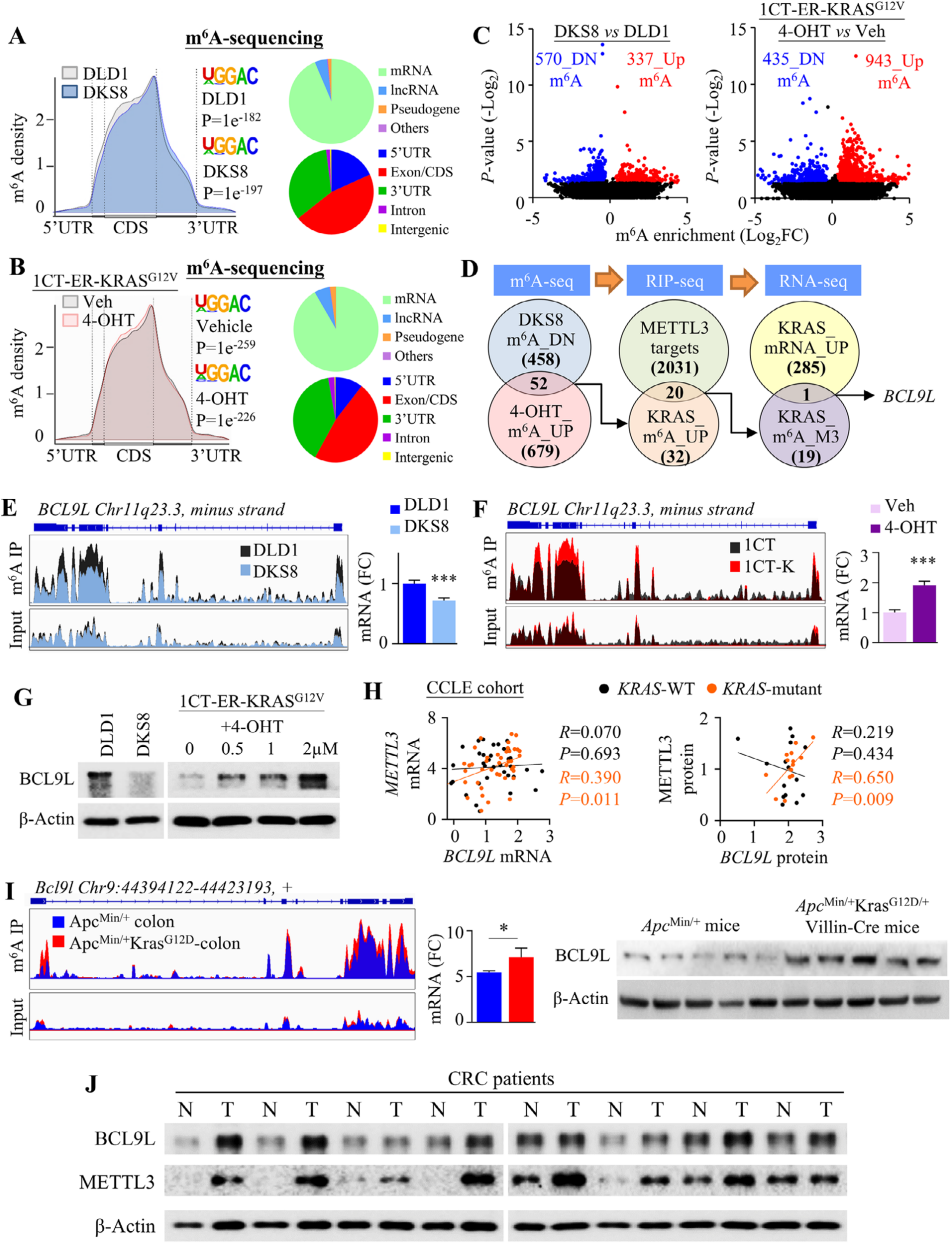
Multiomic analysis identified BCL9L as an m^6^A‐modified target gene in KRAS‐mutant CRC. (A) m^6^A‐seq analysis of DLD1 (KRAS^G13D^), and DKS8 (KRAS^WT^) cells. (B) m^6^A‐seq analysis of 1CT‐ER‐KRAS^G12V^ cells treated with vehicle or 4‐hydroxytamoxifen (4‐OHT, 1 µm) for 72 h. (C) Volcano plot showing differential m^6^A methylated peaks in DKS8 (KRAS^WT^) compared to DLD1 (KRAS^G13D^) cells (*left*). Volcano plot showing differential m^6^A methylated peaks in 1CT‐ER‐KRAS^G12V^ cells treated with 4‐hydroxytamoxifen (4‐OHT, 1 µm) for 72 h compared to vehicle (*right*). (D) Identification of BCL9L as a m^6^A‐modified target in KRAS‐mutant CRC by overlapping m^6^A‐seq (m^6^A peaks down‐regulated in DKS8 vs. DLD1 cells, but up‐regulated in 4‐OHT‐treated 1CT‐ER‐KRAS^G12V^ cells vs. vehicle) (*P* < 0.05; FC>1.3), RIP‐seq (genes interacting with METTL3) (*P* < 0.05; FC>2), and RNA‐seq (genes down‐regulated in DKS8 vs. DLD1 cells, but up‐regulated in 4‐OHT‐treated 1CT‐ER‐KRAS^G12V^ cells vs. vehicle) (*P* < 0.05; FC>1.3) datasets. (E) Integrative Genomics Viewer (IGV) snapshots of Ribo‐seq reads mapping to BCL9L exons in DLD1 (KRAS^G13D^) and DKS8 (KRAS^WT^) cells normalized to input mRNA abundance (*left*). BCL9L mRNA expression (*right*). Student's T‐test. (F) Integrative Genomics Viewer (IGV) snapshots of Ribo‐seq reads mapping to BCL9L exons in 1CT‐ER‐KRAS^G12V^ cells treated with 4‐hydroxytamoxifen (4‐OHT, 1 µm) or vehicle for 72 h, normalized to input mRNA abundance (*left*). BCL9L mRNA expression (*right*). Student's T‐test. (G) Protein expression of BCL9L in DLD1 (KRAS^G13D^) and DKS8 (KRAS^WT^) cells, and in 1CT‐ER‐KRAS^G12V^ cells treated with different doses of 4‐hydroxytamoxifen (4‐OHT, 0.5–2 µm) for 72 h compared to vehicle. (H) Cancer Cell Line Encyclopedia (CCLE) analysis of the correlation between METTL3 and BCL9L at mRNA (*left*) and protein (*right*) levels. KRAS‐wildtype (black) and KRAS‐mutant (orange) cells are indicated in different colors. Pearson R correlation coefficient. (I) Integrative Genomics Viewer (IGV) snapshots of Ribo‐seq reads mapping to BCL9L exons in colon tissues of Apc^Min/+^Kras^G12D/+^ and Apc^Min/+^mice (*left*). BCL9L mRNA (*middle*) and protein (*right*) expression. Student's T‐test. (J) BCL9L and METTL3 protein expression in paired CRC tumor tissues and adjacent normal tissues. ^*^, *p* < 0.05; ^**^, *p* < 0.01; ^***^, *p* < 0.001; ^****^, *p* < 0.0001.

To understand how KRAS‐driven m^6^A modification might play a role in tumorigenesis, we performed m6A‐sequencing in DLD1/DKS8 isogenic cell lines and 1CT‐ER‐KRAS^G12V^ cells treated with vehicle or 4‐OHT. As shown in Figure 4A,B, the normalized distribution of m6A peaks of DLD1 (grey) versus DKS8 (blue) and 1CT‐ER‐KRAS^G12V^ vehicle (grey) vs. 4‐OHT (red) confirmed that mutant KRAS induced m6A levels, particularly in the CDS region. Volcano plots revealed that more m6A peaks were depleted after knockout of mutant KRAS, whereas the majority of m6A peaks were upregulated upon KRAS^G12V^ overexpression (*P* < 0.05; FC>1.3) (Figure 4C). We then performed multiomics integration of m6A‐seq, METTL3‐RIP‐seq, and RNA‐seq (Figure 4D). First, we overlapped 1) m6A peaks down‐regulated in DKS8 versus DLD1 cells; and 2) m6A up‐regulated by 4‐OHT in 1CT‐ER‐KRAS^G12V^ cells. Second, 52 common m6A peaks up‐regulated by mutant KRAS were integrated with METTL3‐binding mRNA in a pulldown assay. Finally, 20 candidates were overlapped with genes whose mRNA was over‐expressed (*P* < 0.05; FC>1.3) in mutant KRAS cells (DLD1 vs DKS8 and 1CT‐ER‐ KRAS^G12V^ Veh. vs 4‐OHT), narrowing down to a single candidate gene, *BCL9L*. The UCSC snapshots of m6A pulldown vs. input verified that mutant KRAS knockout decreased BCL9L m6A level in DKS8 (Figure 4E), together with reduced mRNA expression; whereas activation of KRAS^G12V^ by 4‐OHT induced BCL9L m6A modification and mRNA expression (Figure 4F). Western blot confirmed that mutant KRAS knockout down‐regulated BCL9L, whilst activation of KRAS^G12V^ by 4‐OHT up‐regulated BCL9L protein expression (Figure 4G). Corroborating our findings, METTL3 positively correlated with BCL9L mRNA (*P* < 0.01) and protein (*P* < 0.05) (Figure 4H) levels specifically in KRAS‐mutant CRC cells from Cancer Cell Line Encyclopedia (CCLE), but not in KRAS‐wildtype CRC cells. In vivo, Bcl9l m^6^A modification, mRNA, and protein expression were increased in *Apc* ^Min/+^ *Kras* ^G12D/+^ *Villin*‐*Cre* mice compared to *Apc* ^Min/+^ mice (Figure 4I). METTL3 and BCL9L protein expression were concomitantly up‐regulated in human CRC tissues compared to paired normal tissues (Figure 4J). Taken together, in vitro and in vivo findings indicate BCL9L as a target of mutant *KRAS*‐mediated m^6^A in CRC.

### 2.6 METTL3 Co‐Operates With YTHDF1 to Promote BCL9L Expression in *KRAS*‐Mutant CRC

**FIGURE 5:**
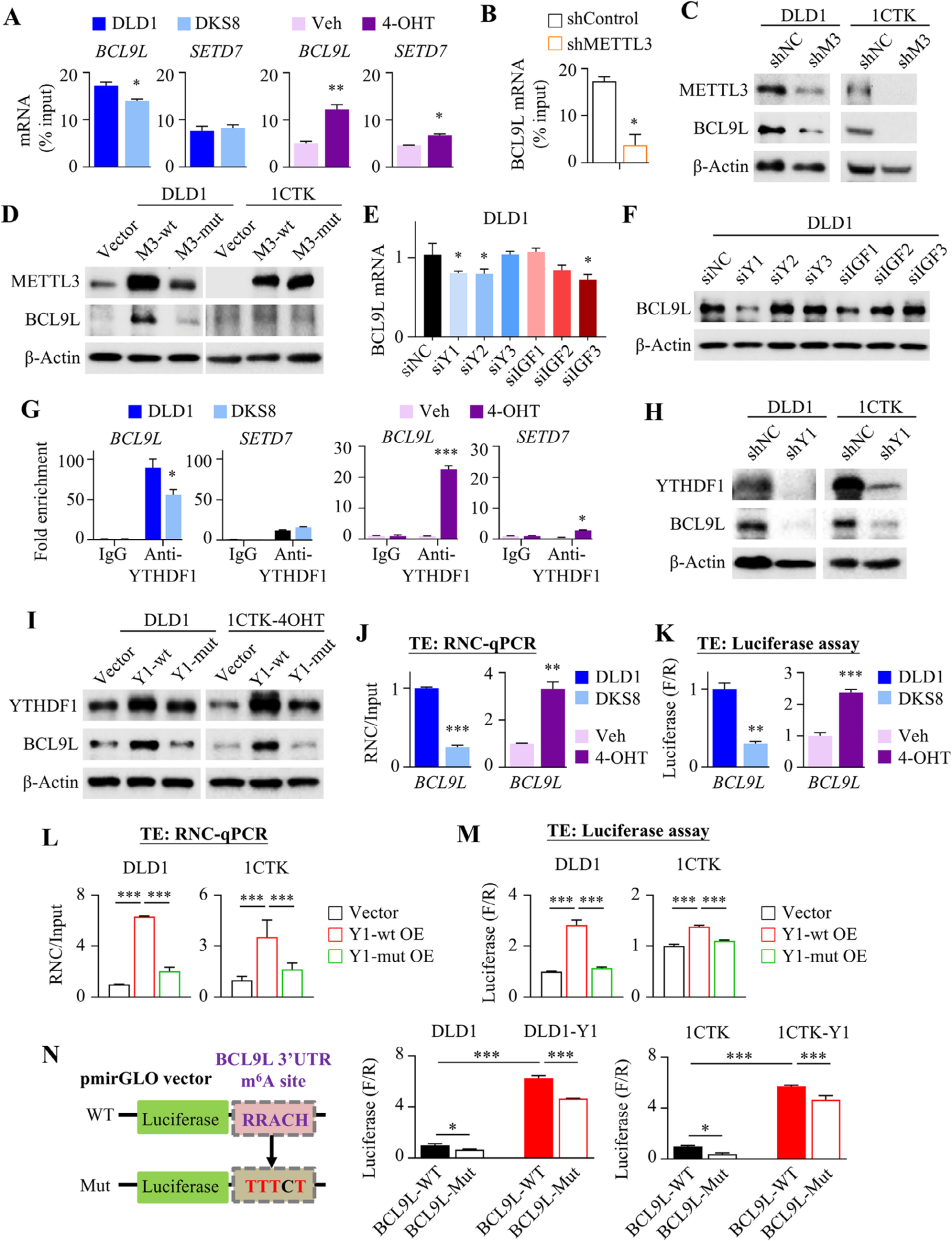
METTL3 and YTHDF1 cooperatively promote the translation and expression of BCL9L in KRAS‐mutant CRC cells. (A) m^6^A‐pulldown qPCR analyses of m^6^A‐modified BCL9L and SETD7 mRNA in DLD1 (KRAS^G13D^) and DKS8 (KRAS^WT^) cells, and in 1CT‐ER‐KRAS^G12V^ cells treated with vehicle or 4‐hydroxytamoxifen (4‐OHT, 1 µm) for 72 h. Student's T‐test. (B) METTL3 knockdown in DLD1 cells significantly reduced BCL9L m^6^A‐modified mRNA levels. Student's T‐test. (C) METTL3 knockdown in CRC cells suppressed BCL9L protein expression. (D) Overexpression of wild‐type METTL3, but not catalytic‐dead mutant METTL3, increased BCL9L expression in CRC cells. (E) Effect of knockdown of m^6^A readers on BCL9L mRNA expression. One‐Way ANOVA. (F) Effect of knockdown of m^6^A readers on BCL9L protein expression. (G) RNA‐immunoprecipitation qPCR (RIP‐qPCR) with anti‐YTHDF1. Binding of BCL9L and SETD7 mRNA was determined in DLD1 (KRAS^G13D^) and DKS8 (KRAS^WT^) cells, and in 1CT‐ER‐KRAS^G12V^ cells treated with vehicle or 4‐hydroxytamoxifen (4‐OHT, 1 µm) for 72 h. Student's T‐test. (H) YTHDF1 knockdown in CRC cells suppressed BCL9L protein expression. (I) Overexpression of wildtype YTHDF1, but not mutant YTHDF1 with m^6^A‐modified mRNA binding motif, increased BCL9L expression in CRC cells. (J) RNC‐qPCR analyses of BCL9L translation efficiency in DLD1 (KRAS^G13D^) and DKS8 (KRAS^WT^) cells, and in 1CT‐ER‐KRAS^G12V^ cells treated with vehicle or 4‐hydroxytamoxifen (4‐OHT, 1 µm) for 72 h. Student's T‐test. (K) Luciferase assays of BCL9L translation efficiency in DLD1 (KRAS^G13D^) and DKS8 (KRAS^WT^) cells, and in 1CT‐ER‐KRAS^G12V^ cells treated with vehicle or 4‐hydroxytamoxifen (4‐OHT, 1 µm) for 72 h. Student's T‐test. (L) RNC‐qPCR analyses of BCL9L translation efficiency in CRC cells expressing wildtype or mutant YTHDF1. One‐Way ANOVA. (M) Luciferase assays of BCL9L translation efficiency in CRC cells expressing wildtype or mutant YTHDF1. One‐Way ANOVA. (N) pmirGLO‐BCL9L‐mutant reporters with mutated m^6^A sites (RRACH to TTTCT) in the 3′‐UTR region demonstrated decreased luciferase activity. One‐Way ANOVA. ^*^, *p* < 0.05; ^**^, *p* < 0.01; ^***^, *p* < 0.001; ^****^, *p* < 0.0001.

We next sought to decipher the molecular mechanisms whereby BCL9L m^6^A modification is up‐regulated in KRAS‐mutant CRC and the consequential effect on its expression. Methylated RNA immunoprecipitation (MeRIP)‐qPCR validated the m^6^A modification levels of BCL9L. As shown in Figure 5A, BCL9L m^6^A modification was decreased in mutant KRAS knockout DKS8 cells compared to DLD1 cells, whereas the addition of 4‐OHT to activate mutant KRAS in 1CTK‐ER‐KRAS^G12V^ cells significantly promoted BCL9L m^6^A modification. Another m^6^A‐modified gene, SETD7, exhibited no differential m^6^A modification by mutant KRAS (Figure 5A). Given that METTL3 is an m^6^A writer up‐regulated in KRAS‐mutant CRC, we next asked if enhanced m^6^A modification by mutant KRAS depends on METTL3. Indeed, knockdown of METTL3 in DLD1 cells reduced BCL9L m^6^A modification (Figure 5B) and BCL9L protein expression (Figure 5C). Whereas ectopic expression of METTL3 increased protein expression of BCL9L in CRC cells, mutant METTL3 lacking m^6^A methyltransferase activity failed to increase BCL9L protein (Figure 5D). These results imply that METTL3 promotes the expression of BCL9L in an m^6^A‐dependent manner.

We next explored m^6^A readers that influence the fate of m^6^A‐modified BCL9L mRNA. To this end, we screened the effect of knockdown of m^6^A readers (siYTHDF1‐3, siIGF2BP1‐3), and determined their effects on BCL9L mRNA and protein expression. As shown in Figure 5E, si‐YTHDF1, siYTHDF2, and siIGF2BP3 moderately suppressed BCL9L mRNA levels. Western blot suggested that siYTHDF1 most profoundly inhibited BCL9L protein expression (Figure 5F). To assess if YTHDF1 interacts with BCL9L mRNA in a mutant KRAS‐dependent fashion, we performed RNA immunoprecipitation (RIP)‐qPCR following pulldown by anti‐YTHDF1. Mutant KRAS knockout decreased interaction between YTHDF1 and BCL9L mRNA (but not SETD7), whereas the addition of 4‐OHT to 1CTK‐ER‐KRAS^G12V^ cells significantly increased BCL9L mRNA pulldown (Figure 5G). Given the documented role of YTHDF1 in facilitating translation of m^6^A‐modified transcripts, we assessed BCL9L protein expression after YTHDF1 knockdown and unraveled that shYTHDF1 decreased BCL9L protein (Figure 5H). Conversely, overexpression of wild‐type YTHDF1 increased BCL9L protein expression, while the m^6^A‐binding defective mutant YTHDF1 had no effect (Figure 5I).

To ascertain if the KRAS‐m^6^A axis boosts BCL9L translation, we performed RNC‐qPCR and luciferase reporter assays. Consistently, BCL9L translation decreased in DKS8 cells compared to DLD1 cells (Figure 5J,K), whilst being increased by 4‐OHT in 1CTK‐ER‐KRAS^G12V^ cells (Figure 5J,K). KRAS‐driven BCL9L translation depends on YTHDF1, as wild‐type YTHDF1, but not mutant YTHDF1, promoted the translation of BCL9L (Figure 5L,M). Furthermore, mutation of BCL9L m^6^A binding sites significantly reduced YTHDF1‐dependent translation according to a luciferase assay (Figure 5N). In summary, we defined that METTL3 and YTHDF1 co‐operatively drive BCL9L expression and translation in *KRAS*‐mutant CRC.

### 2.7 BCL9L Promotes an Immunosuppressive Tumor Microenvironment in KRAS‐Mutant CRC

**FIGURE 6:**
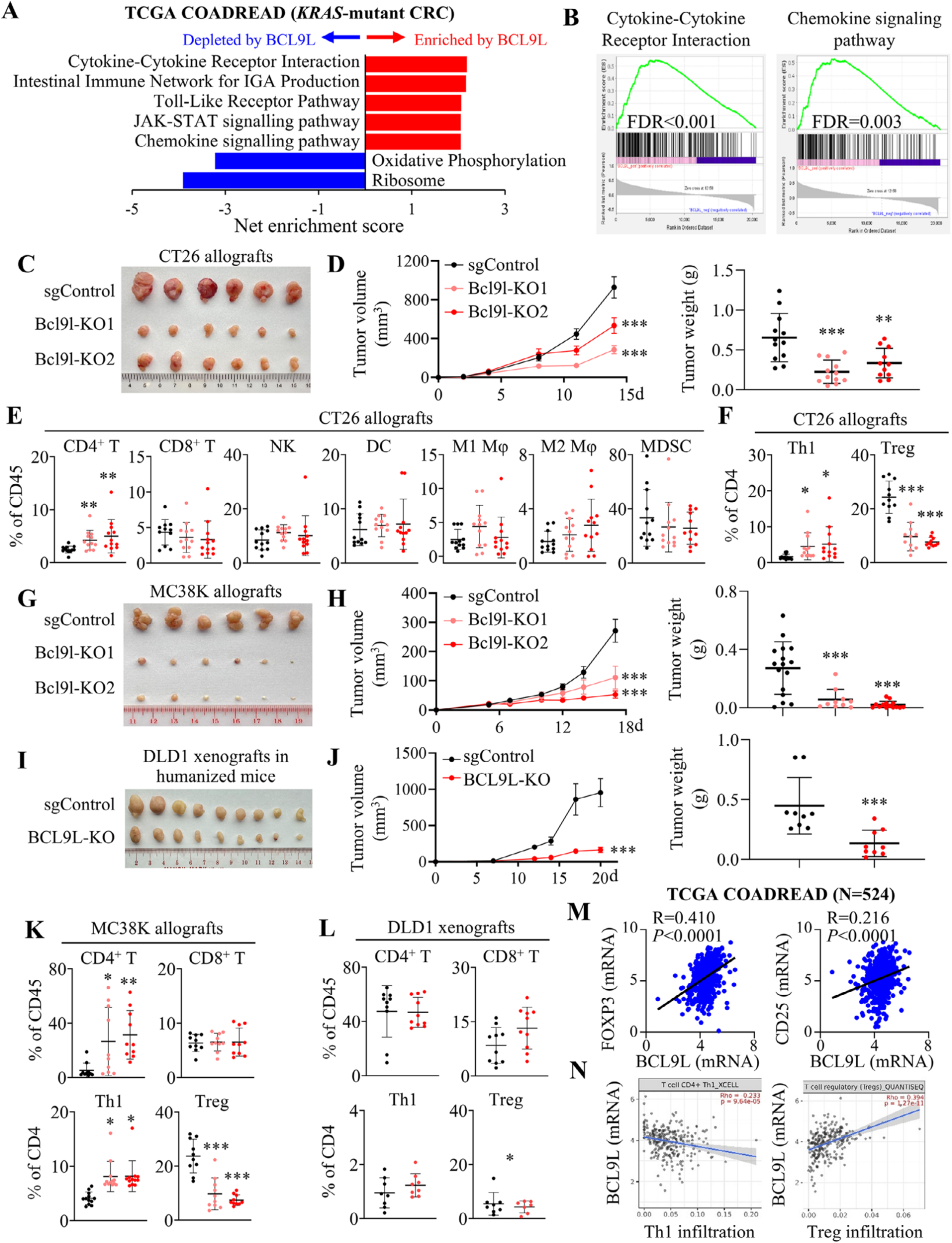
BCL9L promotes KRAS‐mutant CRC growth through modulating the tumor immune microenvironment (A) Gene Set Enrichment Analysis (TCGA) of BCL9L‐associated pathways in KRAS‐mutant CRC patients from the TCGA cohort. (B) GSEA plots showing that BCL9L expression positively correlates with Cytokine‐cytokine receptor interaction and Chemokine signaling pathway in KRAS‐mutant CRC. (C‐D) Effect of BCL9L knockout on the growth of CT26 (KRAS^G12D^) allografts in BALB/c mice. Tumor growth curve: Two‐way ANOVA; Tumor weight: One‐Way ANOVA. (E) Flow cytometry analysis of tumoral immune cells from CT26 (KRAS^G12D^) allografts, with or without BCL9L knockout. One‐Way ANOVA. (F) Flow cytometry analysis of Th1 and T_reg_ cells from CT26 (KRAS^G12D^) allografts, with or without BCL9L knockout. One‐Way ANOVA. (G, H) Effect of BCL9L knockout on the growth of MC38K (KRAS^G12V^) allografts in C57BL/6 J mice. Tumor growth curve: Two‐way ANOVA; Tumor weight: One‐Way ANOVA. (I, J) Effect of BCL9L knockout on the growth of DLD1 (KRAS^G13D^) xenografts in huCD34^+^ humanized NOD/SCID‐γ mice. Tumor growth curve: Two‐way ANOVA; Tumor weight: Student's T‐test. (K) Flow cytometry analysis of CD4^+^ T, CD8^+^ T, Th1 and T_reg_ cells from MC38K (KRAS^G12V^) allografts, with or without BCL9L knockout. One‐Way ANOVA. (L) Flow cytometry analysis of CD4^+^ T, CD8^+^ T, Th1 and T_reg_ cells from DLD1 (KRAS^G13D^) xenografts, with or without BCL9L knockout. Student's T‐test. (M) BCL9L mRNA positively correlates with FOXP3 and CD25 mRNA expression in KRAS‐mutant CRC patients from the TCGA cohort. Pearson R correlation coefficient. (N) BCL9L mRNA expression negatively correlates with Th1 levels but positively correlates with T_reg_ levels in the TCGA CRC cohort. ^*^, *p* < 0.05; ^**^, *p* < 0.01; ^***^, *p* < 0.001; ^****^, *p* < 0.0001.

To interrogate pathways modulated by BCL9L in KRAS‐mutant CRC, we performed Gene Set Enrichment Analysis (GSEA) of 162 KRAS‐mutant CRC samples from the TCGA cohort. Notably, we found that immune‐associated gene sets were enriched in BCL9L‐high tumors, with the cytokine‐cytokine receptor pathway as the top enriched pathway (Figure 6A,B), implying that BCL9L may impact the tumor immune microenvironment (TIME). Concordantly, analyses of the transcriptome of *Apc* ^Min/+^ vs. *Apc* ^Min/+^ *Kras* ^G12D/+^Villin‐Cre mice (Figure S3A) revealed that the top enriched pathways in KRAS‐mutant CRC are highly represented by immune‐related pathways, such as TNF signaling, Cytokine‐cytokine receptor pathway, and IL‐17 signaling (Figure S3A). Corresponding mass cytometry analyses of TIME revealed that mutant KRAS led to the most notable enrichment of T_reg_ in the colon tissues of *Apc* ^Min/+^ *Kras* ^G12D/+^Villin‐Cre as compared to *Apc* ^Min/+^ mice (Figure S3B,C), implying a critical role of mutant KRAS in immune‐related signaling.

Functional studies showed that BCL9L knockdown (Figure S4A) had limited effects on cell proliferation in vitro (Figure S4B,C), as well as tumor growth in immunodeficient mice in vivo (Figure S4D). To decipher if BCL9L mediates its mutant KRAS‐dependent effects via modulating TIME, we established mutant KRAS CRC allografts with or without knockout of BCL9L. In CT26 allografts, BCL9L knockout significantly suppressed tumor growth (Figure 6C,D). Flow cytometry analysis revealed that BCL9L knockout increased infiltration of total CD4^+^ T cells (Figure 6E), whereas other immune cell populations remained unchanged, inferring that BCL9L primarily modulates CD4^+^ T cells in KRAS‐mutant CRC. We dissected CD4^+^ T cell subtypes (Th1, Th2, Th17, and T_reg_), revealing that BCL9L knockout up‐regulated TNF‐α‐ and IFN‐γ‐expressing CD4^+^ T cells (Th1), whilst markedly depleted CD4^+^ T_reg_ (Figure 6F). We next sought to validate the function of BCL9L in modulating tumorigenesis and TIME in KRAS‐mutant CRC. To this end, we generated MC38K allografts in wildtype C57LB/6 mice (Figure 6G,H) and human DLD1 xenografts in CD34^+^‐immune humanized mice (Figure 6I,J). In both models, BCL9L knockout suppressed tumor growth. BCL9L knockout increased CD4^+^ T and Th1 cells while down‐regulating T_reg_ in MC38K tumors (Figure 6K). In DLD1 tumors, total CD4^+^ T cells were unchanged, Th1 cells showed an increasing trend, whereas T_reg_ were significantly down‐regulated (Figure 6L). This phenotypic divergence in DLD1 tumors likely reflects the inherent limitations of humanized mouse systems, which exhibit incomplete functional reconstitution of the human lymphoid lineage compared to syngeneic mouse models. These findings collectively imply that BCL9L knockout suppresses immunosuppressive T_reg_ but re‐invigorates CD4^+^ Th1 cells. Corroborating our experimental observations, analyses of the TCGA dataset revealed positive correlations between BCL9L mRNA and T_reg_ markers FOXP3 and CD25 (Figure 6M). The positive correlation between BCL9L and FOXP3 and CD25 was consistent among MSI‐H and MSS CRC patients, suggesting independence from MSI‐H status (Figure S5A,B). TIMER analyses consistently demonstrated that BCL9L mRNA negatively correlated with Th1 but positively correlated with T_reg_ (Figure 6N). Together, the KRAS‐m^6^A‐BCL9L axis likely plays an important role in modulating the TIME of KRAS‐mutant CRC via T_reg_ cells. We asked whether BCL9L‐dependent cytokines are involved. Cytokine array analysis showed that BCL9L depletion down‐regulated TGF‐β1/2/3, all of which have been shown to promote T_reg_ differentiation (Figure S5C). Supporting this, in the TCGA CRC cohort we observed a positive correlation between TGFB1/2/3 and BCL9L in KRAS‐mutant CRC patients (Figure S5D). In vitro T_reg_ expansion assays also demonstrated that conditioned medium from CT26 cells with BCL9L knockout had an impaired capacity to induce T_reg_ as compared to that from sgControl cells (Figure S5E), an effect rescued by supplementation of TGF‐β1 and abolished by co‐treatment with anti‐TGF‐β. This implies that BCL9L regulates T_reg_ by promoting TGF‐β1 secretion.

### 2.8 Co‐Targeting m^6^A and Mutant KRAS Synergistically Suppressed Tumor Growth

**FIGURE 7:**
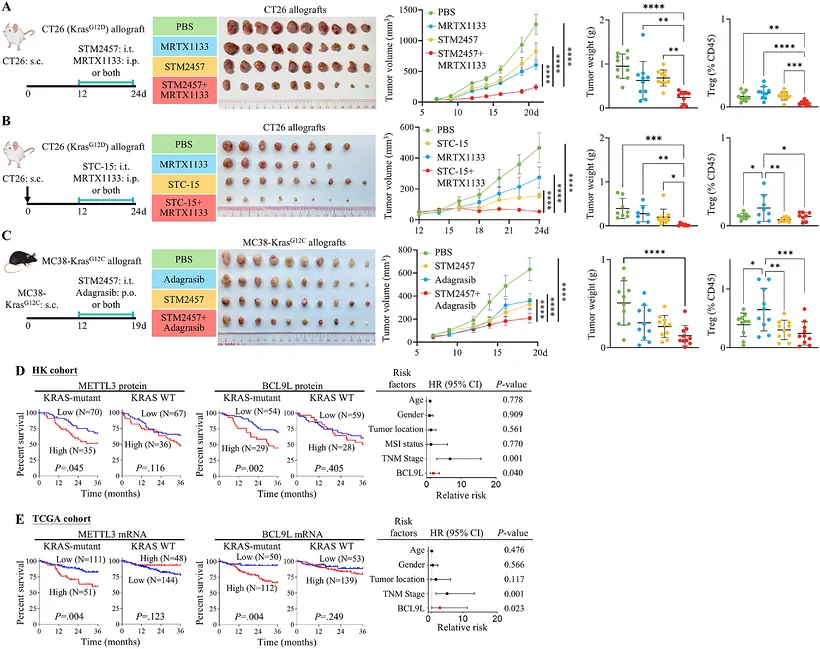
Co‐targeting of METTL3 and mutant KRAS synergistically suppressed tumor growth (A) CT26 (KRAS^G12D^) allografts were established in BALB/c mice, and randomized to receive PBS, STM2457 (250 µg per tumor, i.t.), MRTX1133 (15 mg/kg, i.p.), or STM2457 (250 µg per tumor, i.t.) plus MRTX1133 (15 mg/kg,i.p.). Flow cytometry analysis of T_reg_ cells in CT26 allografts. Tumor growth curve: Two‐way ANOVA; Tumor weight: One‐way ANOVA. (B) CT26 (KRAS^G12D^) allografts were established in BALB/c mice and randomized to receive PBS, STC‐15 (500 µg per tumor, i.t.), MRTX1133 (15 mg/kg, i.p.), or STC‐15 (500 µg per tumor, i.t.) plus MRTX1133 (15 mg/kg, i.p.). Flow cytometry analysis of T_reg_ cells in CT26 allografts. Tumor growth curve: Two‐way ANOVA; Tumor weight: One‐way ANOVA. (C) MC38K (KRAS^G12C^) allografts were established in C57BL/6 J mice, and randomized to receive PBS, STM2457 (250 µg per tumor, i.t.), Adagrasib (25 mg/kg, p.o.), or STM2457 (250 µg per tumor, i.t.) plus Adagrasib (25 mg/kg, p.o.). Flow cytometry analysis of T_reg_ cells in CT26 allografts. Tumor growth curve: Two‐way ANOVA; Tumor weight: One‐way ANOVA. (D) Tissue microarray cohort showed that protein expression of METTL3 (N = 208) and BCL9L (N = 170) predicts poor survival of KRAS‐mutant CRC, but not in KRAS‐wildtype CRC (*left*). Multivariate COX proportional hazards regression analysis of the prognostic value of BCL9L protein in KRAS‐mutant CRC (*right*). (E) TCGA cohort showed that mRNA expression of METTL3 and BCL9L (N = 354) predicts poor survival of KRAS‐mutant CRC, but not in KRAS‐wildtype CRC (*left*). Multivariate COX proportional hazards regression analysis of the prognostic value of BCL9L mRNA in KRAS‐mutant CRC (*right*). (F) Schematic diagram showing the mechanism of action of the mutant KRAS‐m^6^A‐BCL9L axis in promoting CRC progression. ^*^, *p* < 0.05; ^**^, *p* < 0.01; ^***^, *p* < 0.001; ^****^, *p* < 0.0001.

We then determined whether combined targeting of the KRAS‐m^6^A‐BCL9L axis could be a novel strategy for KRAS‐mutant CRC. As there is no specific pharmacological inhibitor for BCL9L, we utilized specific inhibitors of m^6^A writer METTL3. We first tested the effect of STM2547 (METTL3 inhibitor) plus MRTX1133 (KRAS^G12D^ inhibitor) in CT26 (Kras^G12D^) allografts. As shown in Figure 7A, STM2547 or MRTX1133 alone moderately inhibited the growth of CT26 tumors. On the other hand, their combination synergistically suppressed tumor growth, which was significantly stronger than either drug alone (Figure 7A). We further tested a combination of STC15 (METTL3 inhibitor) plus MRTX1133, again showing that the drug combination was synergistic in suppressing tumor growth and leading to tumor regression (Figure 7B). To ask if METTL3 inhibition can act in synergy with clinically approved KRAS inhibitors, we utilized MC38‐KRAS^G12C^ tumor allografts and treated these mice with STM2457, Adagrasib, or their combination. Consistently, combined STM2457 plus Adagrasib was most effective in reducing tumor size and tumor weight (Figure 7C) and was significantly stronger than either drug alone (Figure 7C). Additionally, we measured tumor‐infiltrating T_reg_ cells across the three models. Interestingly, KRAS inhibitors alone elevated T_reg_ cells compared to vehicle control, whilst co‐treatment with METTL3 inhibitors abrogated the increase in T_reg_ cells (Figure 7A–C). The drug combinations did not cause body weight loss and were apparently safe (Figure S6). STC‐15 has completed a Phase I clinical trial, and it was well‐tolerated with mild GI symptoms in ∼15% of the patients [27]. Together, we identified that the combination of METTL3 inhibitor plus KRAS inhibitor synergistically inhibits tumor growth in vivo and represents a promising strategy for boosting KRAS inhibitors' efficacy in CRC.

### 2.9 METTL3 and BCL9L are Prognostic Markers in KRAS‐Mutant CRC

To determine the clinical significance of METTL3 and BCL9L in human KRAS‐mutant CRC, we determined the prognostic significance of these genes. We employed the HK cohort, consisting of tissue microarray samples with *KRAS* mutation information available. As shown in Figure 7D, high METTL3 (*p* = 0.045) or high BCL9L (*p* = 0.002) protein expression was associated with poor survival in KRAS‐mutant CRC; however, neither predicted the survival of KRAS‐wildtype CRC. Multivariate COX regression analysis with age, gender, tumor location, TNM stage, and MSI status as covariates showed that BCL9L protein was an independent factor for poor prognosis in *KRAS*‐mutant CRC patients (*P* < 0.05) (Figure 7D). We next sought to validate our observations in the TCGA dataset (mRNA) consisting of 354 CRC patients. Kaplan‐Meier curves showed that METTL3 (*p* = 0.004) and BCL9L (*p* = 0.004) mRNA expression predicted poor survival in *KRAS*‐mutant CRC (Figure 7E), but not in KRAS‐wildtype CRC. Multivariate COX regression analysis with age, gender, tumor location, and TNM stage as covariates found that BCL9L mRNA is an independent prognostic factor in *KRAS*‐mutant CRC (Figure 7E). MSI status was not included as a covariate in the TCGA dataset, as there are many missing values. KRAS‐m^6^A‐BCL9L axis thus predicts poor survival of *KRAS*‐mutant CRC patients.

## 3 Discussion

Oncogenic mutations in KRAS are highly prevalent in CRC. Nevertheless, the interconnection between mutant KRAS and the epitranscriptome is largely unknown. In this study, we showed that mutant KRAS was associated with elevated m^6^A modification in CRC patients, cell lines, and animal models. Mutant KRAS inhibits autophagy‐mediated METTL3 degradation, thereby increasing METTL3 protein expression. Mechanistically, mutant KRAS promotes a METTL3‐m^6^A‐BCL9L axis to promote intratumoral T_reg_ and subsequent CRC tumorigenesis. Targeting mutant KRAS in combination with a METTL3 inhibitor synergized to suppress tumor growth, representing a promising strategy to boost the efficacy of KRAS inhibitors in CRC.

Among all the commonly occurring genetic mutations, only KRAS mutations demonstrated a positive correlation with m^6^A abundance in CRC patients. Supporting this initial observation, comprehensive evaluation of isogenic CRC cells and transgenic mouse models with or without mutant KRAS validated that expression of mutant KRAS promotes m^6^A mRNA modification, whilst its knockout exerts opposite effects. Whereas most studies have reported the function of m^6^A regulators in catalyzing methylation, demethylation, or binding to m^6^A‐modified mRNA [21, 22, 23, 24], the underlying molecular basis of m^6^A up‐regulation remains unclear. Here, we found a novel mechanism whereby mutant KRAS promotes METTL3 stabilization by suppressing its autophagy‐induced degradation. Autophagy is a conserved pathway for recycling proteins [28]. Traditionally viewed as being non‐selective, emerging evidence indicates that selective autophagy could proceed through the action of selective autophagy receptors, such as p62 [29]. Consistent with the notion that METTL3 is targeted by selective autophagy for degradation, we observed a direct interaction between p62 and METTL3, and that p62 depletion rescued METTL3 protein levels in KRAS‐wildtype CRC cells. Mutant KRAS, by suppressing autophagic flux in CRC cells, thus promotes the stabilization of METTL3 protein levels, which in turn promotes increased m^6^A modification. Previous studies have also reported that suppression of mutant KRAS increased autophagic flux [30, 31] through the action of PI3K. Indeed, mutant KRAS activates p‐mTOR, a master negative regulator of autophagy [32], in CRC cells, whereas mutant KRAS knockout exerts opposite phenotypes. The effect of KRAS mutation on promoting METTL3 expression was consistent among the most prevalent KRAS mutant alleles in CRC (G12D, G13D, G12V), accounting for 75% of all KRAS‐mutant CRC patients. Further, the activation of PI3K‐AKT‐mTOR upstream of p62‐mediated autophagy is a common hallmark shared by most oncogenic KRAS variants. We thus unraveled that mutant KRAS orchestrates increased m^6^A modification in CRC through stabilizing the m^6^A writer METTL3 in vitro and in vivo.

To unveil the potential downstream targets of mutant KRAS‐mediated METTL3 up‐regulation and increased m^6^A modification, we performed integrative RNA‐seq, m^6^A‐seq, and RIP‐seq to identify the direct targets of mutant KRAS‐mediated m^6^A modifications. In line with the mutant KRAS‐METTL3‐m^6^A axis, knockout of mutant KRAS decreased the number of m^6^A peaks in CRC cells, whereas its ectopic expression increased m^6^A‐modified mRNA. Overlap of multi‐omic datasets revealed BCL9L as the downstream target of the mutant KRAS‐METTL3 axis. We showed that mutant KRAS specifically increased m^6^A modification of BCL9L transcripts and its protein expression, an effect reversed by METTL3 knockdown. More specifically, we found that m^6^A modification of BCL9L causes the increased binding of BCL9L mRNA with the m^6^A reader YTHDF1, which then functions to promote the translation of BCL9L. Consistent with our observations, YTHDF1 has been shown to promote the translation of m^6^A‐modified mRNA into proteins in a context‐dependent manner [33, 34]. Hence, the co‐operative effect of METTL3‐mediated m^6^A methylation and YTHDF1‐driven translation underlies the increased BCL9L protein expression in KRAS‐mutant CRC.

BCL9L and its paralog BCL9 are considered to be oncogenic factors that play important roles in tumorigenesis [35]. However, most studies thus far have focused on BCL9 or have studied BCL9/BCL9L in conjunction [36], which have identified them as coactivators of the WNT/β‐catenin signaling pathway. Nevertheless, it is unknown if BCL9L may harbor unique functions independent of BCL9. Intriguingly, mutant KRAS selectively increased m^6^A‐modified BCL9L and its protein expression but had no equivalent effect on BCL9. BCL9L has a notably longer 3’UTR region with a higher number of predicted RRACH motifs (n = 45) compared to BCL9 (n = 16), which may explain its propensity for m^6^A modifications. Mechanistically, we identified that BCL9L is positively correlated with Cytokine‐cytokine receptor signaling, and BCL9L is essential for secretion of TGF‐β isoforms, inferring that BCL9L could independently function to regulate the TIME in the context of KRAS‐mutant CRC. Indeed, while BCL9L knockdown alone had no apparent effects on the growth of KRAS‐mutant CRC cells in vitro and in immunodeficient mice, presumably due to compensatory BCL9L‐driven WNT signals, the loss of BCL9L markedly impaired tumorigenesis in immunocompetent models. Profiling of TIME revealed that BCL9L knockout in KRAS‐mutant CRC significantly altered CD4^+^ T cells, with depletion of immunosuppressive T_reg_ together with the expansion of Th1 cells, thereby skewing the TIME towards an immune‐reactive phenotype. This is consistent with the effect of BCL9L in promoting TGF‐β, a pivotal cytokine that induces FOXP3 in naïve CD4^+^ T cells and drives T_reg_ differentiation [37]. Corroborating our experimental results, BCL9L expression in KRAS‐mutant CRC positively correlated with FOXP3 and CD25 expression, as well as T_reg_ infiltration in the TCGA cohort.

While BCL9L knockout reduced T_reg_ and rescued immunosuppression in KRAS‐mutant CRC, the residual tumor growth in mouse models implies a BCL9L‐independent mechanism. BCL9L likely serves as a major, but not sole, mediator. Among the gene candidates (N = 20) that are METTL3 targets identified by the overlap of m^6^A‐seq and RIP‐seq datasets, several have been implicated in tumorigenesis. For instance, IMCT plays a crucial role in the post‐translational modification of KRAS, which controls its membrane anchoring and activation [38]. E3 ligase RNF40 is upregulated in CRC tumors and has been shown to inhibit apoptosis in CRC cells [39]. SETD1B is a histone methyltransferase that promotes tumor growth and stemness through epigenetic dysregulation [40]. These genes may be auxiliary effectors downstream of the KRAS‐m^6^A axis in promoting CRC.

Given the co‐operative action between mutant KRAS and m^6^A epitranscriptome in promoting CRC, we next evaluated the combination of KRAS inhibitor plus METTL3 inhibitor in mouse models of KRAS‐mutant CRC. Resistance to KRAS inhibitors remains an important issue, in particular for CRC [19, 20]. Here, we showed that METTL3 inhibitors, such as STM2457 and STC‐15, could effectively synergize with KRAS inhibitors to suppress tumor growth. The drug combination was proven effective in the clinically approved KRAS^G12C^ inhibitor Adagrasib and the experimental KRAS^G12D^ inhibitor MRTX1133 [41]. METTL3 inhibitors, e.g., STC‐15, are also undergoing clinical trials, inferring that this drug combination can be of potential translational value. Notably, immunological profiling of the TIME revealed that KRAS inhibitors elevated intratumoral T_reg_. An increased infiltration of T_reg_ has recently been shown to induce resistance to KRAS inhibitors [42]. METTL3 inhibition, by targeting the m^6^A‐BCL9L axis, could return intratumoral T_reg_ to baseline. Combined mutant KRAS plus METTL3 inhibition represents a translatable approach to improve therapy response in KRAS‐mutant CRC. Nevertheless, we have only assessed this drug combination in the context of KRAS^G12D^ and KRAS^G12C^ mutations, and future studies are required to validate the generalizability of this approach across different KRAS isoforms. Furthermore, future evaluation of this drug combination in orthotopic tumor models or genetically engineered mouse models, which better recapitulate the TIME of CRC, will be essential to validate these findings before clinical development.

The impact of these findings was strengthened by the observation that METTL3 and BCL9L were overexpressed in CRC. Further, METTL3 and BCL9L expression at mRNA and protein levels was correlated with poor survival in KRAS‐mutant CRC patients, but not in their wild‐type counterparts. BCL9L expression is also an independent prognostic factor in KRAS‐mutant CRC. Although the hazard ratios of METTL3 and BCL9L were modest, these results inferred the plausible role of METTL3 and BCL9L in promoting KRAS‐mutant CRC.

We would like to acknowledge some of the limitations of this study. In this study, we primarily utilized parental DLD1 and HCT116 cells along with their corresponding mutant KRAS knockout derivatives. A potential limitation of these cell line pairs is that DKS8 and HKE3 cells possess one fewer copy of the *KRAS* gene. To exclude potential confounding effects from copy number differences, future work using monoallelic cell line pairs, such as DKS8 (KRAS ^WT/−^) and DKO1 (KRAS ^G13D/−^), will be invaluable to validate our observations.

In conclusion, we identified mutant KRAS as an important driver of aberrant m^6^A methylation in CRC through protection of METTL3 protein against autophagy‐mediated degradation. This, in turn, promotes m^6^A modification, BCL9L translation, and immunosuppressive TIME. Given the positive circuitry between mutant KRAS and the m^6^A epitranscriptome, co‐targeting of KRAS and METTL3 might improve treatment response in KRAS‐mutant CRC.

## 4 Materials and Methods

### 4.1 Human Patient Samples

The tissue microarray (TMA) cohort was generated from formalin‐fixed, paraffin‐embedded CRC tissues of 208 patients collected at the Prince of Wales Hospital, Hong Kong, with a median follow‐up time of 61.1 months. Among these, 195 cases were MSS CRC patients, among which 98 were KRAS‐mutant CRC. MSI‐H CRC accounts for 13 cases, among which 7 were KRAS‐mutant CRC. Immunohistochemistry was performed using anti‐m^6^A (KHC0143; Proteintech), anti‐METTL3 (15073‐1‐AP; Proteintech, RRID:AB_2142033) or anti‐BCL9L (ab233736; Abcam). TMA scoring was performed by an experienced pathologist (Dr. Alvin Cheung, The Chinese University of Hong Kong) blinded to the nature of the samples. Cases with complete loss of tissue slides or the absence of tumor regions were excluded from analysis. The number of evaluable samples was 208 for METTL3, 175 for m^6^A, and 170 for BCL9L. All the subjects provided informed consent for the study specimens. TCGA COADREAD cohort (Pan‐Cancer normalized data) was downloaded from https://xenabrowser.net/. Samples with mRNA, KRAS mutation status, and survival information available were used for analysis. Informed consent was obtained for all patients, and this study was approved by the Joint Chinese University of Hong Kong‐New Territories East Cluster Clinical Research Ethics Committee (Reference no: 2022.066).

### 4.2 Cell Lines and Transfection

DLD1 (RRID: CVCL_0248), HCT116 (RRID: CVCL_0291) and CT26 (RRID: CVCL_7256) cell lines were acquired from ATCC. DKS8 and HKE3 cells were from Prof. Senji Shirasawa (Fukuoka University, Japan). 1CT normal colon cells were obtained from Prof. Jerry Shay (UT Southwestern Medical Center, TX). 1CT cells were transduced with lentiviral‐KRAS^G12V^ to provide 1CT‐K and with lentiviral‐ER‐KRAS^G12V^ to provide 1CT‐ER‐ER‐KRAS^G12V^ cells. All experiments were performed with cells that underwent 10 passages of thawing. All cells, except 1CT cells, were cultured in DMEM (Gibco, 11965118) supplemented with 10% (v/v) fetal bovine serum (Gibco, 16140071) and antibiotic‐antimycotic (Gibco, 15240112) at 37 °C and 5% CO_2_. 1CT cells and their derivatives were cultured as described [5]. Plasmids for METTL3‐wt, METTL3‐mut, YTHDF1‐wt, and YTHDF1‐mut have been described previously [21, 43]. BCL9L knockout was performed with the KN2.0 CRISPR knockout kit (KN502116; Origene) according to the manufacturer's instructions. siBCL9L was obtained from ThermoFisher (s49113, s49115).

### 4.3 Dot Blot Assay

Total RNA was extracted from cells or tissues using Trizol reagent (ThermoFisher), and mRNA was purified by Dynabeads mRNA Purification Kit (ThermoFisher). After quantification using a NanoDrop, mRNA was spotted onto a nitrocellulose membrane at indicated concentrations. After drying in a 60°C oven for 30 min, the RNA blot was exposed to ultraviolet light for 30 min. After blocking in 5% bovine serum albumin in PBS‐T, the blot was incubated with anti‐m^6^A (1:1000, ab151230, Abcam, RRID: AB_2753144) overnight at 4°C, followed by incubation with secondary antibodies and visualization by Clarity Western ECL Substrate (GE Healthcare). For loading control, replicate blots were stained in Methylene Blue staining solution.

### 4.4 Liquid Chromatography‐Mass Spectrometry (LC‐MS)

Total RNA was extracted from cells or tissues using Trizol reagent (ThermoFisher), and mRNA was purified by Dynabeads mRNA Purification Kit (ThermoFisher). Isolated RNA was then digested with the nucleoside digestion mix (M0649S, New England Biolabs) at 37°C for 18 h, followed by heating at 80°C for 3 min to quench the reaction. LC‐MS (Dionex Ultimate 3000 UPLC, RRID: SCR_019840, ThermoFisher, Waltham, MA, USA) was utilized for nucleotide analysis, and the m^6^A‐to‐A ratio was determined.

### 4.5 *Apc* ^Min/+^ *Kras* ^G12D/+^ *Villin*‐*Cre* Mouse Model

*Apc* ^Min/+^ *Kras* ^G12D/+^ *Villin*‐*Cre* mice were provided by Nanjing Biomedical Research Institute, Nanjing University. At 7 weeks of age, the *Apc* ^Min/+^ *Kras* ^G12D/+^ *Villin*‐*Cre* and *Apc* ^Min/+^ mice (RRID: IMSR_JAX:002020) were sacrificed, and paired CRC and adjacent normal tissues were snap‐frozen and stored at −80°C. RNA was extracted from frozen tissues for downstream analysis. No significant weight loss (> 15%) was observed during the study, and tumor volume did not exceed 2000 mm^3^. All the animal studies were performed in accordance with ARRIVE guidelines and approved by the Animal Experimentation Ethics Committee (AEEC), The Chinese University of Hong Kong (No: 22‐044‐GRF).

### 4.6 *Kras* ^G12D/+^ *Cdx2‐Cre* ^ERT2^ Mouse Model

Transgenic colon‐specific Kras knock‐in mice (*Kras* ^G12D/+^ *Cdx2‐Cre* ^ERT2^) were purchased from Nanjing Biomedical Research Institute, Nanjing University. Eight‐week‐old *Kras* ^G12D/+^ *Cdx2‐Cre* ^ERT2^ mice and wildtype littermates were injected with 4 injections of tamoxifen (100 mg/kg, i.p.) to activate mutant KRAS, followed by harvesting 2 weeks after the first injection. Baseline tissue samples were collected separately on the day of the first injection. No significant weight loss (> 15%) was observed during the study. All animal studies were performed in accordance with ARRIVE guidelines and approved by AEEC, The Chinese University of Hong Kong (No: 22‐044‐GRF).

### 4.7 *Mettl3* ^KI^ *Cdx2‐Cre* ^ERT2^ Mouse Model

Transgenic colon‐specific Kras knock‐in mice (*Mettl3* ^KI^ *Cdx2‐Cre* ^ERT2^) were purchased from Cyagen (Suzhou, China). Eight‐week‐old *Mettl3* ^KI^ *Cdx2‐Cre* ^ERT2^ mice and wildtype littermates were injected with 4 injections of tamoxifen (100 mg/kg, i.p.) to induce METTL3 knock‐in, followed by harvesting 180 days after the first injection to collect colon tissues for DNA extraction and KRAS sequencing. No significant weight loss (> 15%) was observed during the study. All animal studies were performed in accordance with ARRIVE guidelines and approved by AEEC, The Chinese University of Hong Kong (No: 22‐044‐GRF).

### 4.8 Western Blot

Whole cell proteins were extracted using Cytobuster protein extraction reagent supplemented with complete mini protease inhibitor cocktail (Roche). Proteins were separated by SDS‐PAGE and transferred onto PVDF membranes. After blocking with 5% BSA for 1 h, blots were probed with primary antibodies overnight at 4°C, followed by incubation with secondary antibodies and visualization by Clarity Western ECL Substrate (GE Healthcare).

### 4.9 Real‐Time Quantitative PCR

Total RNA was extracted using Trizol (ThermoFisher), and cDNA was synthesized using a high‐capacity cDNA reverse transcription kit (ThermoFisher). qPCR was performed using TB Green Premix Ex Taq (Takara) in a QuantStudio Flex 7 real‐time PCR system (ThermoFisher, RRID: SCR_020245). The primers used are listed in Table S1.

### 4.10 Immunofluorescence

Cells on glass coverslips were fixed with ice‐cold 4% paraformaldehyde, permeabilized with 0.5% Triton X‐100 in PBS, and blocked with PBS containing 1–2% BSA at room temperature. Cells were incubated with primary antibody overnight at 4°C, followed by secondary antibodies for 1 h at 37°C. The slides were washed with 0.05% Tween‐20 in PBS and mounted with DAPI‐containing medium. Images were acquired using a fluorescence microscope (TCS SP8, Leica). Anti‐p62 (ab280086; Abcam, RRID:AB_2923299) and anti‐METTL3 (15073‐1‐AP; Proteintech, RRID:AB_2142033) were used for staining. For the LC3B‐GFP‐mCherry assay, cells were transfected with the pCDH‐EF1a‐mCherry‐EGFP‐LC3B plasmid (RRID: Addgene_170446) for 72 h and then examined by confocal microscopy.

### 4.11 Co‐Immunoprecipitation (co‐IP) and Mass Spectrometry

For co‐IP, 1 mg of DLD1 lysates was incubated with 1 µg anti‐p62 or IgG overnight at 4°C, followed by pulldown with 50 µl Protein A/G Mix Magnetic Beads (Merch Millipore) for 1 h. The beads were washed three times with RIPA buffer, and proteins were eluted by heating in SDS‐PAGE denaturing buffer. The pulldown lysates were separated by SDS‐PAGE and then stained using silver staining (Pierce Silver Stain Kit; ThermoFisher). Major bands were excised and trypsin‐digested for proteomic analyses by LC‐MS/MS (Biotree). Raw MS files were processed using Proteome Discoverer software and proteins identified by matching to the UniProt database.

### 4.12 Surface Plasmon Resonance Binding Assay

Surface plasmon resonance (SPR) was performed in a Biacore T200 biosensor (GE Healthcare) to study the binding affinity. Human recombinant p62 protein was immobilized on a sensor chip CM5 (Biacore T200, GE Healthcare) with an amine coupling kit. Different concentrations of METTL3 in HEPES buffer were introduced through a microflow system, with an association time of 90 s followed by dissociation for 200 s. All binding experiments were performed at 25°C, and the binding affinity was determined by Biacore T200 evaluation software 3.1 (RRID: SCR_019718).

### 4.13 Cross‐Linking Mass Spectrometry and Molecular Docking

To study the interaction between METTL3 and p62, recombinant proteins were mixed in a 1:1 ratio, and the crosslinker (Bis) was added at 25°C for 30 min. Afterwards, 20 mm Tris‐HCl was added to quench the crosslinker. The proteins were then separated by SDS‐PAGE, digested, and analyzed by LC‐MS/MS (Orbitrap Exploris 480 Mass Spectrometer, RRID: SCR_027000). For molecular docking, the protein structures of p62 (ID: AF‐P51681‐F1) and METTL3 (ID: AF‐Q86U44‐F1) were obtained from the UniProt database. Docking was performed in the HDOCK software, and the contact site was referenced to the cross‐linking results. Pymol was used to identify the binding amino acid residues and to visualize protein interactions.

### 4.14 M^6^A‐Sequencing and M^6^A‐qPCR

Total RNA was extracted with TRIzol, and DNA contamination was removed using DNase, followed by RNA fragmentation in 10 mmol/L Tris‐HCl and 10 mmol/L ZnCl_2_ for 5 min at 70°C. The fragmented RNA was immunoprecipitated by anti‐m^6^A (Abcam, ab208577, RRID: AB_2916290) and Protein A/G Magnetic beads after incubation at 4°C for 6 h. The beads were then washed with low‐salt IP buffer (50 mm NaCl, 10 mm Tris‐HCl, pH 7.5, 0.1% IGEPAL CA‐630) and high‐salt IP buffer (500 mm NaCl, 10 mm Tris‐HCl, pH 7.5, 0.1% IGEPAL CA‐630). RNA was eluted using RLT buffer (Qiagen) and purified. Immunoprecipitated RNA and input RNA were reverse transcribed using a high‐capacity cDNA reverse transcription kit and sequenced on the NovaSeq platform for m^6^A‐seq, and qPCR using gene‐specific primers.

### 4.15 RNA‐Immunoprecipitation qPCR (RIP‐qPCR) and RIP‐Seq

RIP was performed using anti‐YTHDF1 antibody (26787‐1‐AP; Proteintech, RRID: AB_2880635) and EZ‐Magna RIP RNA‐Binding Protein Immunoprecipitation Kit. Briefly, cells were lysed with RIP Lysis buffer and incubated with magnetic beads bound with the anti‐YTHDF1 antibody overnight at 4°C. After repeated washing (6x), RNA was released by proteinase K digestion in 1% SDS at 55°C for 30 min. RNA was isolated and reverse‐transcribed and analyzed by qPCR. RIP‐seq data for METTL3 was obtained from [44].

### 4.16 Ribosome‐Nascent Chain Complex qPCR (RNC‐qPCR)

Cells were treated with 100 mg/mL of CHX for 15 min, rinsed with PBS, and lysed in ribosome buffer (20 mm HEPES‐KOH, pH 7.4, 15 mm MgCl_2_, 200 mm KCl, 100 mm CHX, and 2 mm dithiothreitol) containing 1% Triton X‐100. After 30 min on ice, cell lysates were collected and centrifuged. Supernatants were transferred into 10 mL of 30% sucrose in RB buffer. Ribosome‐nascent chain complexes (RNC) were pelleted after ultracentrifugation at 185 000 g for 5 hat 4°C. Total RNA and RNC‐RNA were respectively isolated by using TRIzol reagent. Isolated RNAs were reverse‐transcribed and analyzed by qPCR.

### 4.17 Luciferase Reporter Assay

Cells were co‐transfected with pmiRGLO‐BCL9L‐ 3’UTR or pmiRGLO‐BCL9L‐Mut‐3’UTR plasmid, together with pRL‐TK plasmid. After 24 h, cells were lysed and analyzed with Dual‐Glo Luciferase Assay (Promega). Luciferase (F‐luc) was normalized to Renilla (R‐luc).

### 4.18 Murine CRC Allograft Models With BCL9L Knockout

CT26‐sgControl and CT26‐sgBCL9L cells (5 × 10^5^ cells/tumor) were subcutaneously injected into male BALB/c mice (6‐8 weeks old, RRID: IMSR_JAX:000651). MC38K (Kras^G12V^)‐sgControl and MC38K‐sgBCL9L cells (5 × 10^5^ cells/tumor) were subcutaneously injected into male C57BL/6 J mice (6‐8 weeks old, RRID: IMSR_JAX:000664). Tumor size was measured by a digital caliper 2–3 times a week and tumor volume was calculated as follows: volume = length × width^2^ × 0.5. No significant weight loss (> 15%) was observed during the study, and tumor volume did not exceed 2 000 mm^3^. At harvest, tumor weight was measured, and tumor tissues were processed for flow cytometry or stored at −80°C. All animal studies were performed in accordance with ARRIVE guidelines and approved by AEEC, The Chinese University of Hong Kong (No: 22‐044‐GRF).

### 4.19 Drug Treatment Models

CT26 cells (5 × 10^5^ cells/tumor) were subcutaneously injected into male BALB/c mice (6‐8 weeks old), while MC38K (Kras^G12C^) cells (5 × 10^5^ cells/tumor) were subcutaneously injected into male C57BL/6 J mice (6‐8 weeks old). After tumor establishment, mice were randomized and treated as follows. For Figure 7A, 1) vehicle; 2) MRTX1133 (15 mg/kg, i.p. daily); 3) STM2457 (250 µg per tumor, i.t. every other day), or 4) STM2457 (250 µg per tumor, i.t., every other day) plus MRTX1133 (15 mg/kg, i.p. daily). For Figure 7B, 1) vehicle; 2) MRTX1133 (15 mg/kg, i.p. daily); 3) STC‐15 (500 µg per tumor, i.t., every other day), or 4) STC‐15 (500 µg per tumor, i.t., every other day) plus MRTX1133 (15 mg/kg, i.p. daily). For Figure 7C, 1) vehicle; 2) Adagrasib (25 mg/kg, p.o. daily); 3) STM2457 (250 µg per tumor, i.t. every other day), or 4) STM2457 (250 µg per tumor, i.t. every other day) plus Adagrasib (25 mg/kg, p.o. daily). Tumor size was measured by a digital caliper 2–3 times a week and tumor volume was calculated as follows: volume = length × width^2^ × 0.5. No significant weight loss (> 15%) was observed during the study, and tumor volume did not exceed 2 000 mm^3^. At harvest, tumor weight was measured, and tumor tissues were processed for flow cytometry or stored at −80°C. All animal studies were performed in accordance with ARRIVE guidelines and approved by AEEC, The Chinese University of Hong Kong (No: 22‐044‐GRF).

### 4.20 CD34^+^ Humanized Mouse Model

CD34^+^ humanized mice were used at week 16 post‐injection of CD34^+^ hematopoietic stem cells as described [24] and implanted with DLD1‐sgControl and DLD1‐sgBCL9L cells (1 × 10^6^ cells/tumor). Tumor size was measured by a digital caliper 2–3 times a week and tumor volume was calculated as follows: volume = length × width^2^ × 0.5. No significant weight loss (> 15%) was observed during the study, and tumor volume did not exceed 2,000mm^3^. At harvest, tumor weight was measured, and tumor tissues were processed for flow cytometry or stored at ‐80°C. All animal studies were performed in accordance with ARRIVE guidelines and approved by AEEC, The Chinese University of Hong Kong (No: 22‐044‐GRF).

### 4.21 Flow Cytometry Analysis

After harvesting mice, tumor samples were dissected and digested with a solution containing 1% bovine serum albumin (BSA), 0.5 mg/mL collagenase D (Roche, Basel, Switzerland), and 0.25 mg/mL DNase I (Roche) at 37°C for 1 h. Next, the samples were filtered through a 70‐µm cell strainer to obtain a single cell suspension for flow cytometry. For intracellular cell target staining, the cells were incubated for 4 h at 37°C with Cell Stimulation Cocktail (eBioscience; 00‐4975‐93) in complete RPMI 1640 medium (Gibco) for cytokine stimulation. For surface target staining, the cells were resuspended in cell staining buffer (Biolegend; 420201) mixed with specific antibodies for 0.5 h at 4°C. The intracellular antigens were captured by specific antibodies for 2 h at 4°C after using fixation and permeabilization buffer (eBioscience; 00‐5523‐00). Finally, the cell suspension was collected through a 40‐µm cell strainer. Flow cytometry analysis was conducted with a Northern Lights instrument (Cytek, RRID: SCR_027072) or a FACS Celesta analyzer (BD, RRID: SCR_019597). Data were analyzed with FlowJo V10 (RRID: SCR_008520). The antibodies used are listed in Table S2.

### 4.22 In Vitro T_reg_ Expansion Assay

Naive CD4^+^ T Cells were isolated from mouse spleen using Naive CD4^+^ T Cell Isolation Kit (BioLegend; 480040) following the manufacturer's protocol. Isolated CD4^+^ cells were seeded at 1 × 10^5^ cells per well in 96‐well plate and cultured in complete DMEM medium containing 50% (v/v) conditioned media from CT26‐sgControl, CT26‐sgBCL9L‐1, and CT26‐sgBCL9L‐2 cells plus anti‐mouse CD3, CD28, and CD2 (STEMCELL; 100–1572) and murine IL‐2 (10 ng/mL) (STEMCELL; 78081) for 5 days. To confirm the role of TGF‐β in CM‐induced T_reg_ polarization, recombinant human TGF‐β1 (5 ng/mL) (MCE; HY‐P7117) or TGF‐β monoclonal antibody (5 ng/mL) (ThermoFisher; MA5‐23795, RRID: AB_2609812) was added. Expansion of T_reg_ was determined by flow cytometry analysis.

### 4.23 Statistical Analysis

All data are presented as mean±standard deviation (S.D.). Comparisons between 2 groups were performed using two‐tailed, unpaired Student's T‐test or Mann‐Whitney U test; and for those with more than 3 groups, one‐way ANOVA was performed with Fisher's LSD test post‐hoc analysis. Survival was estimated by the Kaplan‐Meier method and the log‐rank test. All the above statistical analyses were performed in GraphPad Prism 9 (RRID: SCR_002798). Multivariate Cox regression analysis was performed with SPSS. P‐values < 0.05 were considered significant.

## Author Contributions

D.C., P.H., R.T., Q.W., L.H.K., F.J. and L.Z. performed experiments. H.C. provided expertise in data analysis. W.K. and K.F.T. provided clinical samples and conducted the histological examination. J.Y. supervised the study and reviewed the manuscript. C.C.W. designed and supervised the study and revised the manuscript. All authors have read and approved the final manuscript.

## Funding

This project was supported by the National Nature Science Foundation of China (82504203, 2023ZD0501400, 2023ZD0500200), Research Grants Council (RGC)‐General Research Fund (14101322, 14101725), RGC‐Collaborative Research Fund (C4008‐23WF, C4042‐24GF), and Health and Medical Research Fund (08190706, 22210122)

## Ethical Declaration

This study was approved by the Joint Chinese University of Hong Kong‐New Territories East Cluster Clinical Research Ethics Committee (Reference no: 2022.066) and complied with the ethical standards set out in the Declaration of Helsinki. Informed consent was obtained from all patients. All animal studies were performed in accordance with ARRIVE guidelines and approved by AEEC, The Chinese University of Hong Kong (No: 22‐044‐GRF).

## Conflicts of Interest

The authors declare no conflicts of interest.

## Supporting information




**Supporting File**: advs77627‐sup‐0001‐SuppMat.pdf.

## Data Availability

The data supporting this study's findings are available from the corresponding author upon reasonable request
