# Supplementary material for: Mutant KRAS‐m6A Epitranscriptome Axis Promotes Colorectal Cancer and is a Therapeutic Target
Source: Adv Sci (Weinh). 2026 Sep 9:e77627. Online ahead of print. doi: 10.1002/advs.77627 (PMC13558909; doi:10.1002/advs.77627)
Supplement: Supplementary file 1 [file ADVS-9999-e77627-s001.pdf]

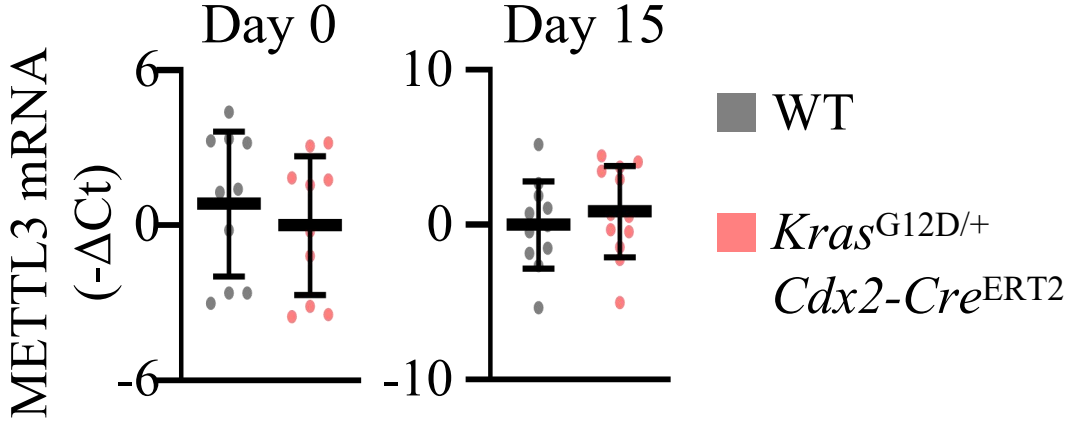

Figure S1. METTL3 mRNA expression in *Kras*$^{G12D/+}$*Cdx2-Cre*^{ERT2} mouse colon and their wildtype counterparts.

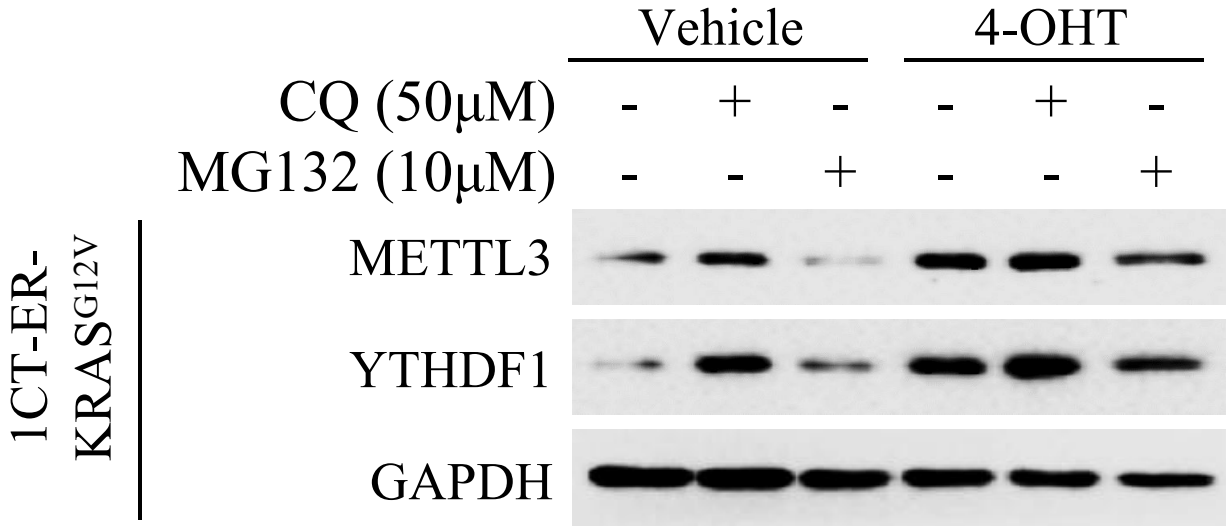

Figure S2. The effect of CQ and MG132 on METTL3 protein expression in 1CT-ER-KRAS^{G12V} cells treated with vehicle or 4-OHT for 72 h (1 μM).

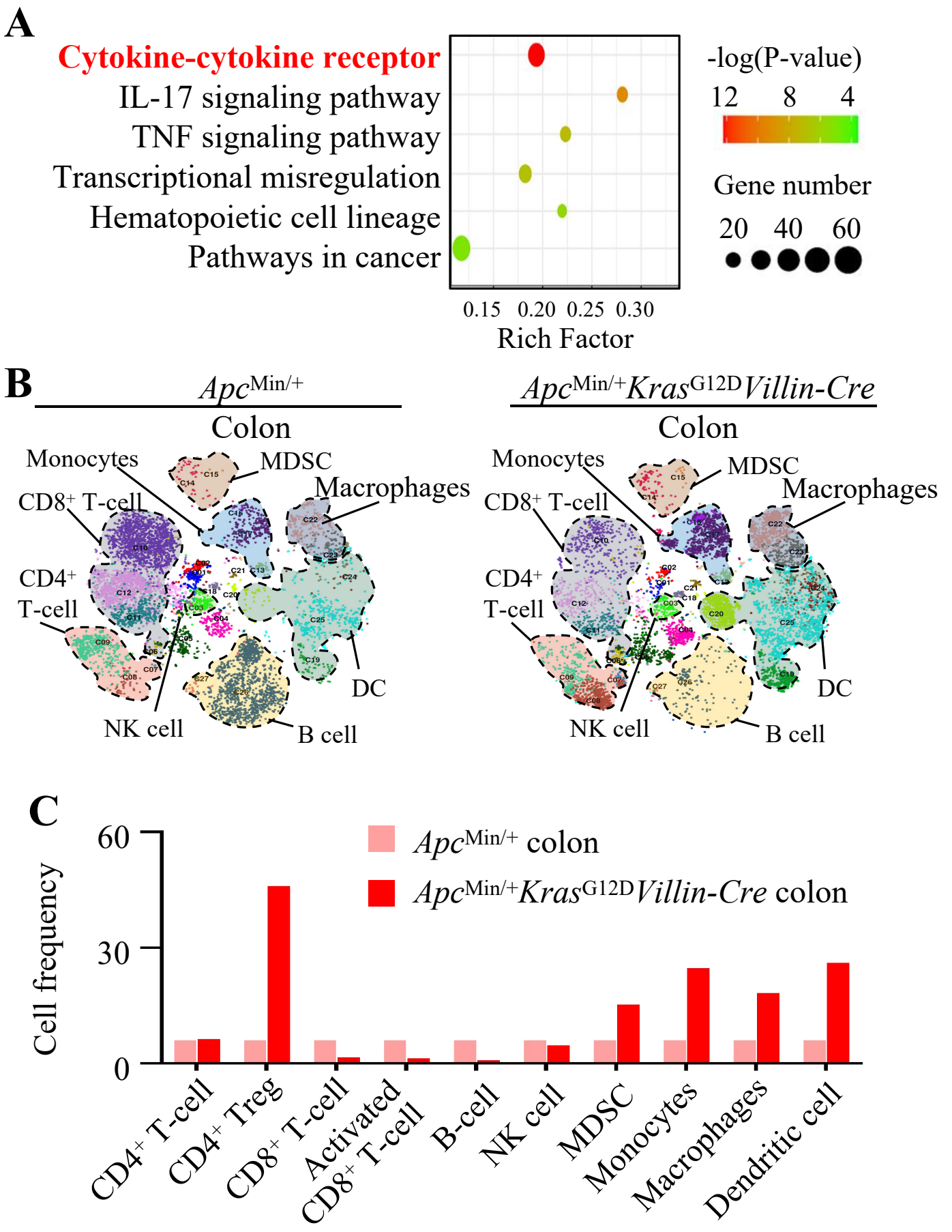

Figure S3. (A) GSEA of pathways enriched in *Apc*$^{Min/+}$*Kras*$^{G12D/+}$*Villin-Cre* mouse colon compared to *Apc*$^{Min/+}$ mouse colon as assessed by RNA-seq. **(B)** Mass cytometry of tumor infiltrating immune cells in *Apc*$^{Min/+}$*Kras*$^{G12D/+}$*Villin-Cre* mouse colon compared to *Apc*$^{Min/+}$ mouse colon. **(C)** Frequency of different immune cell types as assessed by mass cytometry.

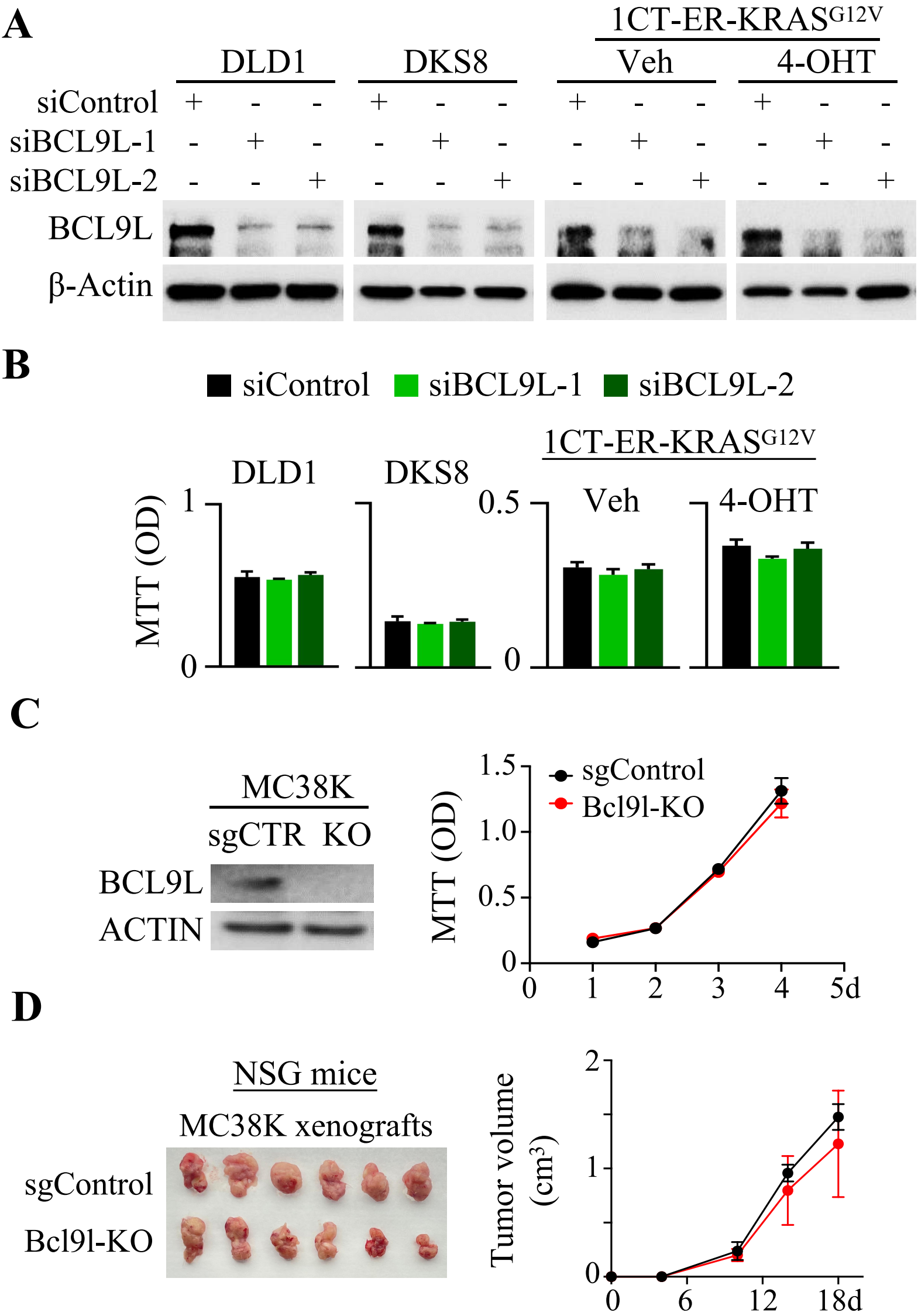

Figure S4. (A) BCL9L knockdown in different cell lines. **(B)** BCL9L knockdown had no effect on cell growth. **(C)** BCL9L knockout in MC38 (KRAS^{G12V}) cells had no effect on *in vitro* cell growth. **(D)** BCL9L knockout in MC38 (KRAS^{G12V}) cells had no effect on tumor growth in immunodeficient mice.

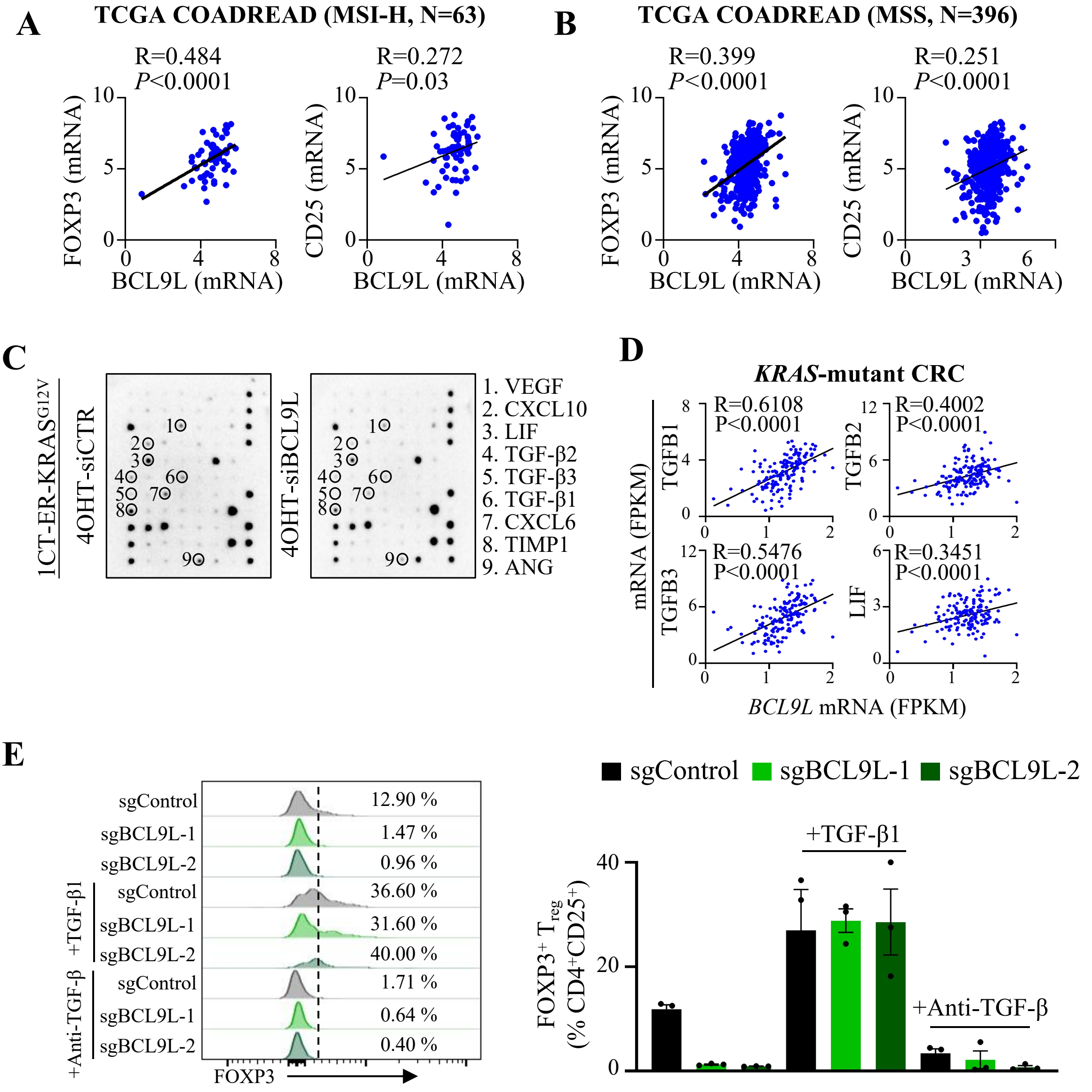

Figure S5. (A) Correlation between BCL9L with FOXP3 and CD25 in microsatellite instability high (MSI-H) CRC in TCGA CRC cohort. **(B)** Correlation between BCL9L with FOXP3 and CD25 in microsatellite stable (MSS) CRC in TCGA CRC cohort. **(C)** Antibody array analysis of secreted cytokines from control and BCL9L knockdown in 1CT-ER-KRAS^{G12V} cells treated with 4-OHT for 72 h (1 μM). **(D)** Correlation between mRNA expression of BCL9L and key cytokines in the TCGA CRC cohort. **(E)** Effect of CT26-sgControl, CT26-sgBCL9L-1, and CT26-sgBCL9L-2 conditioned medium on Treg expansion *in vitro*.

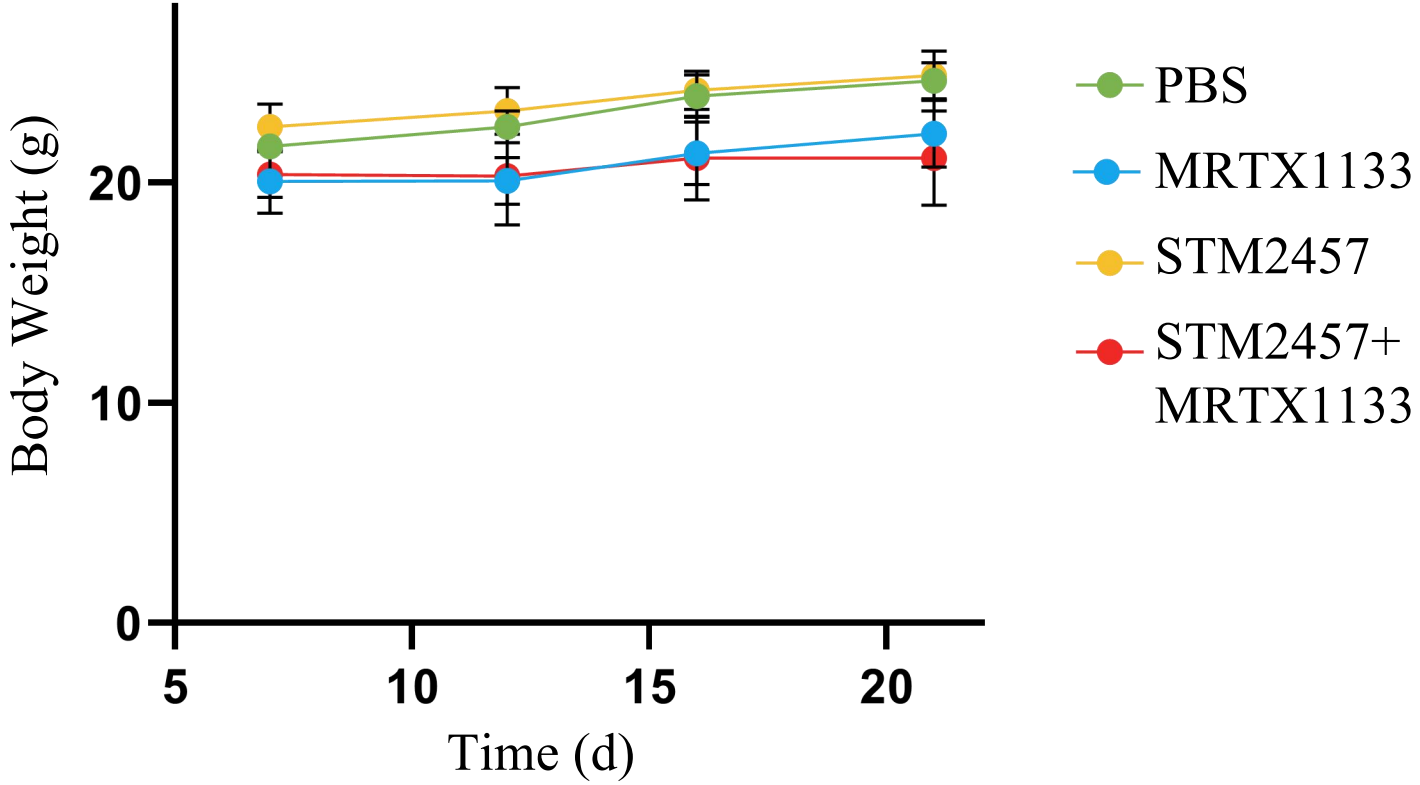

Figure S6. Body weight of mice subjected to the combination treatment in Figure 7A.

Table S1. Primers used in this study

Primer	sequence 5'-3'
m-kras1-F	ATGACTGAGTATAAACTTGT
m-kras1-R	TCGTACTCATCCACAAAGTG
m-kras2-F	TACAGGAAACAAGTAATTGATGGAGAA
m-kras2-R	ATAATGGTGAATATCTTCAAATGATTTAGT
m-GAPDH-F	CATCACTGCCACCCAGAAGACTG
m-GAPDH-R	ATGCCAGTGAGCTTCCCGTTCAG
h-B-actin-F	CACCATTGGCAATGAGCGGTTC
h-B-actin-R	AGGTCTTTGCGGATGTCCACGT
m-BCL9L-F	GGCCGGGCTGGTATTAAGAT
m-BCL9L-R	GTGGGGTAACCTCGCACC
h-BCL9L-F	CACAATGCCATCAAGACCATC
h-BCL9L-R	AGTTCAGGTGCATCTGGCTG

Table S2. Antibodies used for flow cytometry analysis

**Antibodies**		
violetFluor 500 anti-mouse CD45	TONBO	Cat#: 85-0451; RRID: AB_3695656
APC-Cyanine7 anti-mouse CD3	TONBO	Cat#: 25-0032; RRID: AB_2621619
FITC anti-mouse CD4	Biolegend	Cat#: 100510; RRID: AB_312713
Brilliant Violet 711 anti-mouse CD8a	Biolegend	Cat#: 100748; RRID: AB_2562100
Brilliant Violet 785 anti-mouse NK1.1	Biolegend	Cat#: 156539; RRID: AB_3097464
Brilliant Violet 605 anti- mouse CD279 (PD-1)	Biolegend	Cat#: 135219; RRID: AB_11125371
Brilliant Violet 421 anti-mouse FOXP3	Biolegend	Cat#: 126419; RRID: AB_2565933
PE anti-mouse CD25	Biolegend	Cat#: 113704; RRID: AB_2927943
Brilliant Violet 605 anti- mouse CD103	Biolegend	Cat#: 121433: RRID: AB_2629724
APC anti-mouse TCR γ/δ Antibody	Biolegend	Cat#: 118116; RRID: AB_1731824
PerCP/Cyanine5.5 anti-mouse CD19	Biolegend	Cat#: 152406; RRID: AB_2629815
PE/Cyanine7 anti-mouse IFN-γ	Biolegend	Cat#: 505826; RRID: AB_2295770
Brilliant Violet 650 anti-mouse TNF-α	Biolegend	Cat#: 506333; RRID: AB_2562450
APC anti-human/mouse Granzyme B	Biolegend	Cat#: 372204; RRID: AB_2687028
PE anti-human/mouse CD11b	Biolegend	Cat#: 101208; RRID: AB_312791
APC/Fire 810 anti-human/mouse CD11b	Biolegend	Cat#: 101288; RRID: AB_2910274
PE/Cyanine7 anti-mouse F4/80	Biolegend	Cat#: 123114; RRID: AB_892478
Brilliant Violet 421 anti-mouse CD206	Biolegend	Cat#: 141717; RRID: AB_2562232
PE/Cyanine7 anti-mouse CD11c	Biolegend	Cat#: 117318; RRID: AB_493568
PerCP anti-mouse Ly-6G/Ly-6C (Gr-1)	Biolegend	Cat#: 108426: RRID: AB_893558

redFluor 710 Anti-Mouse MHC Class II	Cytek	Cat#: 80-5321 RRID: AB_2621997
PE/Dazzle 594 anti-mouse IL-4	Biolegend	Cat#: 504132: RRID: AB_2564559
Brilliant Violet 785 anti-mouse IL-17A	Biolegend	Cat#: 506928: RRID: AB_2629787
Alexa Fluor 647 anti-mouse IL-22	Biolegend	Cat#: 516406: RRID: AB_2280206
PE/Fire 640 anti-mouse CD185 (CXCR5)	Biolegend	Cat#: 145549; RID: AB_3106150
Brilliant Violet 570 anti-mouse CD62L	Biolegend	Cat#: 104433; RID: AB_10900262
Brilliant Violet 750 anti-mouse/human CD44	Biolegend	Cat#: 103079; RID: AB_2941373
Spark Red 718 anti-mouse CD69	Biolegend	Cat#: 104559; RID: AB_2924442
Brilliant Violet 605 anti-human CD45	Biolegend	Cat#: 304042; RRID: AB_2562106
PE anti-human CD3	Biolegend	Cat#: 317308; RRID: AB_571913
Brilliant Violet 421 anti-human CD8	Biolegend	Cat#: 344747; RRID: AB_2629583
PE/Cyanine5 anti-human CD4	Biolegend	Cat#: 317411; RRID: AB_571956
FITC anti-human TNF-α	Biolegend	Cat#: 502906; RRID: AB_315258
Brilliant Violet 711 anti-human IFN-γ	Biolegend	Cat#: 502540; RRID: AB_2563506
Spark Red 718 anti-human CD279 (PD-1)	Biolegend	Cat#: 621633; RRID: AB_3106105
Brilliant Violet 785 anti-human FOXP3	Biolegend	Cat#: 320135; RRID: AB_3106176
Brilliant Violet 650 anti-human IL-17A	Biolegend	Cat#: 512345; RRID: AB_3683467
PE/Cyanine7 anti-human IL-4	Biolegend	Cat#: 500823; RRID: AB_2126746
Brilliant Violet 711 anti-human CD14	Biolegend	Cat#: 301838; RRID: AB_2562909
Brilliant Violet 785 anti-human CD68	Biolegend	Cat#: 333826; RRID: AB_2800880
Spark PLUS B550 anti-human CD86	Biolegend	Cat#: 305222; RRID: AB_2564407
PE/Fire 700 anti-human CD206	Biolegend	Cat#: 321153; RRID: AB_2910389

PE anti-human CD19	Biolegend	Cat#: 302207; RRID: AB_314237
Brilliant Violet 421 anti-human CD56	Biolegend	Cat#: 362551; RRID: AB_2566060
APC anti-human CD33	Biolegend	Cat#: 366605; RRID: AB_2565753
Brilliant Violet 510 anti-human CD16	Biolegend	Cat#: 302047; RRID: AB_2561380
PE/Cyanine7 anti-human HLA-DR	Biolegend	Cat#: 307615; RRID: AB_493588
